# Crimean-Congo haemorrhagic fever virus in ticks, domestic, and wild animals

**DOI:** 10.3389/fvets.2024.1513123

**Published:** 2025-01-16

**Authors:** Seyma S. Celina, Jignesh Italiya, Allan Obonyom Tekkara, Jiří Černý

**Affiliations:** Center for Infectious Animal Diseases, Faculty of Tropical AgriSciences, Czech University of Life Sciences Prague, Prague, Czechia

**Keywords:** Crimean-Congo haemorrhagic fever virus, ticks, livestock, wildlife, zoonotic disease

## Abstract

Crimean-Congo haemorrhagic fever virus (CCHFV) poses a significant public health threat due to its potential for causing severe disease in humans and its wide geographic distribution. The virus, primarily transmitted by *Hyalomma* ticks, is prevalent across Africa, Asia, Europe, and the Middle East. Understanding the virus’s spread among tick populations is crucial for assessing its transmission dynamics. Vertebrates play a key role in CCHF epidemiology by supporting tick populations and acting as virus carriers during viremia. Livestock, such as cattle, sheep, and goats, amplify the virus and increase tick numbers, posing zoonotic risks. Wildlife, while asymptomatic, can serve as reservoirs. Birds generally do not show signs of the virus but can introduce infected ticks to new regions. This review compiles information on CCHFV’s tick vectors and vertebrate hosts, emphasizing their roles in the virus’s transmission dynamics. Understanding these dynamics is essential for developing effective control and prevention strategies.

## 1 Introduction

Crimean-Congo haemorrhagic fever virus (CCHFV) is a lipid-enveloped, single-stranded RNA virus in the *Orthonairovirus* genus (*Nairoviridae* family). It causes Crimean-Congo haemorrhagic fever (CCHF) in humans, a severe disease with significant public health implications due to its widespread prevalence. CCHF is among the most widely distributed tick-borne viral diseases, endemic across Africa, Asia, Eastern and Southern Europe, and the Middle East, with case fatality rates ranging from 5 to 40% (1–3).

*Hyalomma* ticks, particularly *Hyalomma marginatum*, are the primary vectors for CCHFV. They feed on various domestic ruminants (e.g., sheep, goats, and cattle) and wild animals (e.g., hares, hedgehogs, certain rodents, and ostriches) (4). Ticks play a crucial role in spreading the virus to humans through bites or direct contact with infected animal tissues. Infected vertebrates, although asymptomatic, sustain tick populations and facilitate CCHFV transmission during viremia (5).

**Figure 1:**
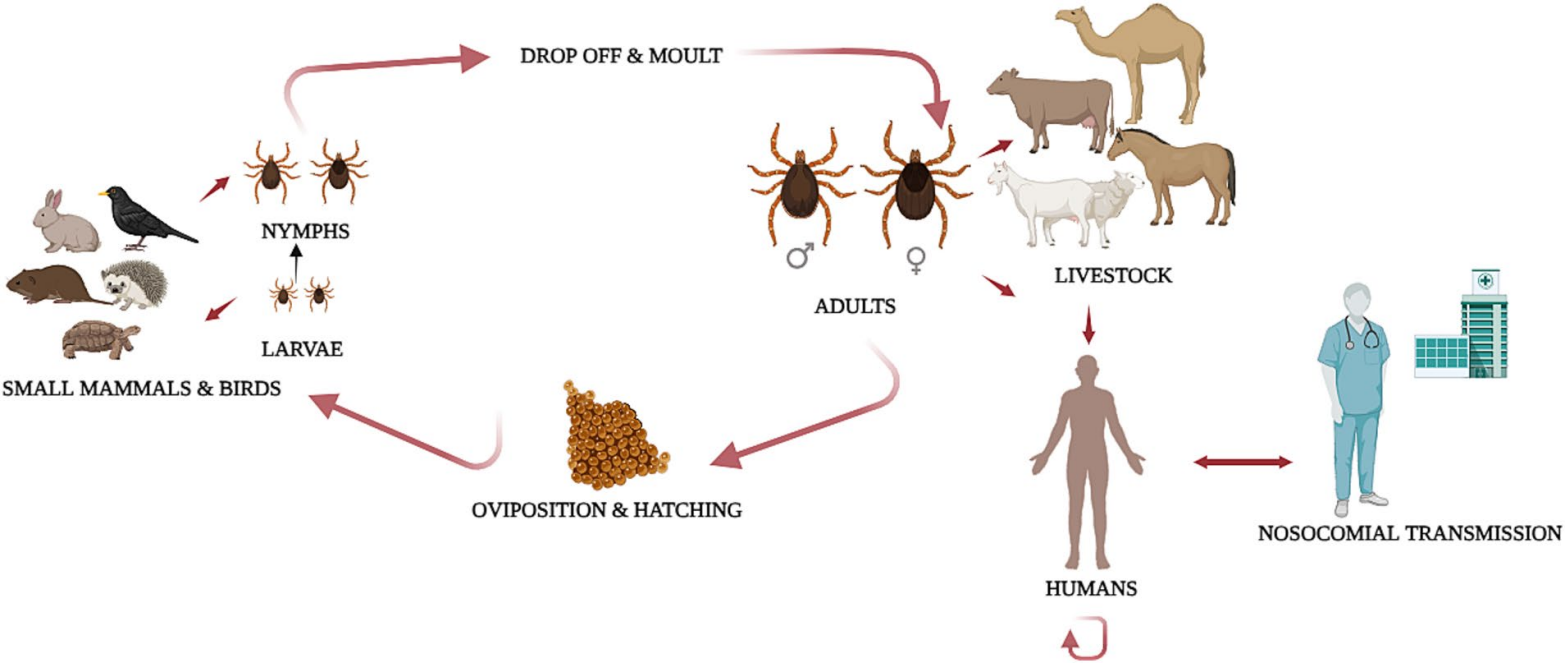
Life cycle of *Hyalomma marginatum* and transmission route of Crimean-Congo haemorrhagic fever virus (CCHFV). *Hyalomma marginatum* is a two-host tick. Upon hatching, larvae seek small animal hosts, such as birds, lagomorphs or rodents, for their first blood meal. After engorgement, the larvae molt into nymphs while remaining on the same host. The nymphs then continue feeding on the same animal until they engorge and drop off to molt into adults. Adult ticks seek larger vertebrate hosts, such as livestock, for feeding and mating. Engorged females then detach to oviposit in the environment. CCHFV transmission occurs between ticks and vertebrate hosts and through co-feeding between ticks. Humans can become infected through tick bites, contact with infected animal fluids, or nosocomial transmission. Secondary human-to-human transmission occurs through direct exposure to the blood, bodily fluids, organs, or secretions of infected individuals. The original figure was created with BioRender (https://Biorender.com/).

Small mammals, such as hares and hedgehogs, amplify immature ticks, while larger domestic animals, including cattle, goats, and sheep, host adult ticks (Figure 1). Although CCHFV has a short viremia in small mammals, their role in CCHFV ecology is significant, as population surges, especially among hares, are linked to disease outbreaks (6, 7). Large domestic mammals inadvertently expose humans to CCHFV, especially during slaughter (8–12). Birds, except for ostriches, generally do not show viremia but may carry infected ticks to new regions (7).

Serological evidence confirms CCHFV exposure in various domestic and wild animals, with experimental infections validating their susceptibility (5). Understanding the virus’s persistence within tick populations, their role as vectors, and the factors influencing viral transmission is essential for effective control strategies. Examining CCHFV in livestock, which often serve as amplifying hosts, provides insights into the virus’s impact on animal health and potential spillover to humans. Additionally, studying CCHFV within wildlife populations is essential for understanding its broader epidemiology.

This review compiles information on CCHFV tick vectors and vertebrate hosts, focusing on their roles in virus transmission and providing a comprehensive resource for understanding CCHFV in animals.

## 2 CCHFV in animals

### 2.1 CCHFV in ticks

The first documented outbreak of CCHF was reported in the Crimean region of the former Soviet Union in 1944, where 200 military personnel suffered from an acute febrile illness with haemorrhagic symptoms, resulting in a 10% fatality rate (1). Investigating the situation, a team led by Mikhail Chumakov found that tick exposure caused these cases. Collecting over 3,000 blood-sucking arthropods, they observed an abundance of ticks, particularly the *H. marginatum* species, now recognized as the primary CCHFV vector (7). These infections were linked to abandoned cultivated lands during the German occupation, enabling tick host expansion. Subsequently, the virus was independently recognized as the cause of illness in the Congo in 1969, leading to the name Crimean-Congo Haemorrhagic Fever Virus (7). Since then, comprehensive studies have consistently reaffirmed ticks as the primary transmission source and reservoir for CCHFV in nature.

CCHFV infection persists throughout the tick life cycle without detrimental effects, allowing the virus to survive transstadially and vertically. Although the frequency of transstadial transmission, the percentage of infected eggs, and the number of generations that can sustain the virus are not well understood. However, ticks can survive for extended periods without feeding, which supports the overwintering of CCHFV, allowing them to act as reservoirs even when vertebrate hosts are absent (13).

Ticks of the Ixodidae family, especially those of the genus *Hyalomma*, are considered both as reservoirs and vectors for CCHFV. *Hyalomma marginatum* has the most prominent role globally in the natural history of CCHF in the Old World. Dramatic increases in the circulation of CCHFV coincide with significant expansions in *H. marginatum* populations, driven by favorable weather conditions and human-induced ecological alterations (14, 15).

Altough the virus is transmitted mainly by tick species in the *Hyalomma* genus, CCHFV has been isolated from other ticks belonging to the genera *Amblyomma*, *Dermacentor*, *Haemaphysalis*, and *Rhipicephalus*. However, there is limited evidence indicating the active circulation of CCHFV among non-*Hyalomma* tick species in natural transmission cycles (16).

**Figure 2:**
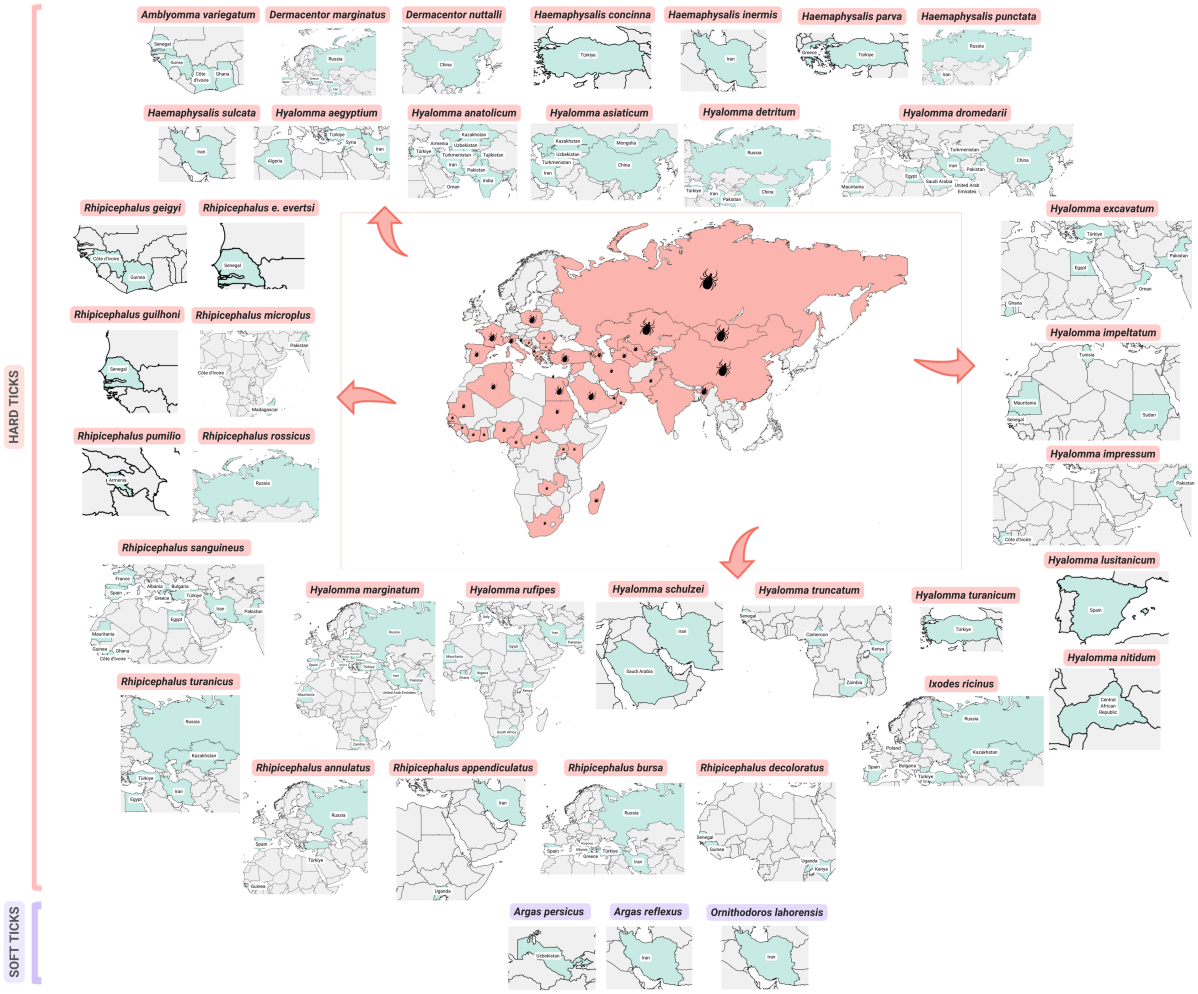
Geographic distribution of Crimean-Congo haemorrhagic fever virus detection in ticks.

CCHFV has been reported in 39 tick species collected from a variety of hosts (13, 16). These include one species from *Amblyomma*, two species from *Dermacentor*, 15 species from *Hyalomma*, five species from *Haemaphysalis*, one species from *Ixodes*, 12 species from *Rhipicephalus*, and three species from the Argasidae family within the genera *Argas* and *Ornithodoros* (Table 1; Figure 2). This wide range of tick species highlights the potential role of numerous ticks in both spreading and maintaining the virus across various regions and host ecosystems.

**Table 1:** List of tick species infected by Crimean-Congo haemorrhagic fever virus.

Order	Family	Scientific name	Country	References
Ixodida	Ixodidae	*Amblyomma variegatum*	Côte d’Ivoire, Ghana, Guinea, Senegal	(19, 90, 92–94)
		*Dermacentor marginatus*	Greece, Iran, Russia, Spain, Türkiye	(95–102)
		*Dermacentor nuttalli*	China	(103)
		*Haemaphysalis concinna*	Türkiye	(104)
		*Haemaphysalis inermis*	Iran	(105)
		*Haemaphysalis parva*	Greece, Türkiye	(102, 106)
		*Haemaphysalis punctata*	Iran, Russia	(95, 105)
		*Haemaphysalis sulcata*	Iran	(107)
		*Hyalomma aegyptium*	Algeria, Iran, Syria, Türkiye	(64, 108–110)
		*Hyalomma anatolicum*	Armenia, India, Iran, Kazakhstan, Pakistan, Oman, Tajikistan, Türkiye, Turkmenistan, Uzbekistan	(17, 39, 99, 104, 105, 107, 111–125)
		*Hyalomma asiaticum*	China, Iran, Kazakhstan, Mongolia, Turkmenistan, Uzbekistan	(103, 105, 126–132)
		*Hyalomma detritum* – (syn. *Hyalomma scupense*)	China, Iran, Pakistan, Russia, Türkiye	(95, 99, 103–105, 111, 115)
		*Hyalomma dromedarii*	China, Egypt, Iran, Mauritania, Pakistan, Saudi Arabia, Turkmenistan, United Arab Emirates	(105, 111–114, 128, 129, 133–137)
		*Hyalomma excavatum*	Egypt, Ghana, Oman, Pakistan, Türkiye	(90, 98, 111, 124, 135)
		*Hyalomma impeltatum*	Mauritania, Senegal, Sudan, Tunisia	(94, 138–140)
		*Hyalomma impressum*	Côte d’Ivoire, Pakistan	(93, 113)
		*Hyalomma lusitanicum*	Spain	(96)
		*Hyalomma nitidum*	Central African Republic	(141)
		*Hyalomma marginatum*	Albania, Bosnia, Bulgaria, Iran, Kosovo, Mauritania, Romania, Pakistan, Russia, Spain, Türkiye United Arab Emirates, Zambia	(20, 95, 104, 105, 108, 111, 113, 114, 127, 142–150)
		*Hyalomma rufipes*	Egypt, Ghana, Iran, Italy, Kenya, Mauritania, Nigeria, Pakistan, Senegal, South Africa	(16, 19, 94, 113, 139, 151–153, 224)
		*Hyalomma schulzei*	Iran, Saudi Arabia	(105, 108, 134)
		*Hyalomma truncatum*	Cameroon, Kenya, Senegal, South Africa, Zambia	(94, 144, 151, 154, 224)
		*Hyalomma turanicum*	Türkiye	(104)
		*Ixodes ricinus*	Bulgaria, Kazakhstan, Kosovo, Poland, Russia, Spain, Türkiye	(18, 20, 95–97, 99, 130, 155)
		*Rhipicephalus (Boophilus) annulatus*	Guinea, Russia, Spain, Türkiye	(92, 95, 96, 143)
		*Rhipicephalus appendiculatus*	Iran, Uganda	(127, 156)
		*Rhipicephalus bursa*	Albania, Greece, Iran, Kosovo, Russia, Spain, Türkiye	(95, 104, 106, 108, 143)
		*Rhipicephalus (Boophilus) decoloratus*	Guinea, Kenya, Senegal, Uganda	(92, 94, 157, 158)
		*Rhipicephalus e. evertsi*	Senegal	(94, 159)
		*Rhipicephalus geigyi*	Côte d’Ivoire, Guinea	(92, 93)
		*Rhipicephalus guilhoni*	Senegal	(159)
		*Rhipicephalus microplus*	Côte d’Ivoire, Madagascar, Pakistan	(93, 113)
		*Rhipicephalus pumilio*	Armenia	(120)
		*Rhipicephalus rossicus*	Russia	(95)
		*Rhipicephalus sanguineus*	Albania, Bulgaria, Côte d’Ivoire, Egypt, France, Greece, Iran, Ghana, Guinea, Mauritania, Pakistan, Spain, Türkiye	(16, 92, 93, 97, 105, 107, 114, 115, 117–119, 125, 139, 147, 160–163)
		*Rhipicephalus turanicus*	Egypt, Iran, Kazakhstan, Russia, Türkiye	(18, 95, 98–100, 104, 115, 126, 127, 162)
	Argasidae	*Argas persicus*	Uzbekistan	(120)
		*Argas reflexus*	Iran	(115)
		*Ornithodoros lahorensis*	Iran	(108)

Detection of CCHFV in ticks predominantly relies on reverse transcriptase-polymerase chain reaction (RT-PCR) due to its high specificity and sensitivity in amplifying viral RNA. Additionally, a few studies employed immunological methods, including enzyme-linked immunosorbent assay (ELISA) [e.g., studies by (17, 18)], immunofluorescence assay (IFA) (19), and a combination of indirect hemagglutination fluorescence assay (IHFA) with RT-PCR (20).

*Hyalomma marginatum* is recognized as the primary vector in the Old World. Among the tick species found infected with CCHFV, 15 are confirmed vectors, while 16 are considered potential vectors (16). In addition to *H. marginatum*, confirmed vectors of CCHFV include *Amblyomma* var*iegatum*, *H. aegyptium, H. anatolicum*, *H. asiaticum, H. asiaticum kozlovi*, *H. detritum, H. dromedarii*, *H. excavatum, H. impeltatum, H. rufipes*, *H. schulzei*, *H. truncatum, H. turanicum* and *R. bursa*. This classification is based on documented infection rates, infection records, and observations across over 30 tick species. Potential vectors include *D. marginatus, D. nuttalli, Ha. concinna, Ha. inermis, Ha. parva, Ha. punctata, Ha. sulcata, I. ricinus, R. annulatus, R. appendiculatus, R. decoloratus, R. evertsi evertsi, R. geigyi, R. guilhoni, R. sanguineus,* and *R. turanicus* (16).

Detecting a virus within an arthropod does not necessarily mean it is an active vector (13). Studies on the vector competence of ticks for CCHFV reveal that ixodid (hard) ticks, particularly those in the *Hyalomma* genus, are highly susceptible to infection and can transmit the virus through biting. Conversely, argasid (soft) ticks are generally not efficient CCHFV vectors (13). The evolutionary dynamics of CCHFV are closely constrained by the necessity to maintain high adaptability within both arthropod and vertebrate host environments. To validate a tick species as a vector, several steps are necessary: the ticks must feed on naturally infected hosts without artificial virus exposure, the virus must be detected in the ticks after molting, and the infected ticks must then feed on naïve hosts. The virus should then be found in these hosts and subsequently in the new generation of ticks from the initially infected adults. Strict adherence to these procedures is essential for accurately determining the vectorial abilities of specific tick species. However, these experiments are particularly challenging to perform because CCHFV is a biosafety level 4 (BSL-4) pathogen, requiring high-level containment facilities for safety.

Further studies are needed to evaluate the vector competence of various tick species for CCHFV transmission and to explore factors influencing the spread of the virus. Understanding both vector competence and vectorial capacity—the extent of transmission—is essential for predicting CCHFV’s spread into new areas. Surveillance of the virus in ticks is an effective tool for monitoring the virus’s introduction or circulation within vulnerable populations. This surveillance helps assess human exposure risk, identify high-risk areas, and establish early warning systems for potential outbreaks. This surveillance is essential not only for accumulating information about pathogen epidemiology but also for clarifying the role of CCHFV tick vectors in public and veterinary health (16).

### 2.2 Serological detection of CCHFV in animals

Serological detection of CCHFV in animals provides crucial information about its ecological role. Because CCHFV often causes a short-lived viremia and can be asymptomatic, directly detecting the virus can be difficult. Thus, serological surveys are essential for monitoring the spread of CCHFV in animal populations and assessing spillover risk to humans.

Common serological methods include ELISA, IFA, and neutralization tests. These techniques help identify animals exposed to the virus, even when symptoms are absent or the infection is not active (21).

ELISA is the most frequently used method for detecting anti-CCHFV antibodies across various animal species. This technique typically targets the nucleocapsid protein (NP) of the virus (22). However, because the Hazara virus (HAZV) and CCHFV belong to the same serogroup, their NPs are genetically similar, leading to cross-reactivity in tests. Studies have shown that sera from animals vaccinated with HAZV can weakly cross-react with CCHFV in immunofluorescence and immunoblot assays, although commercial CCHFV ELISAs used in field studies generally do not show this cross-reactivity (23). Similarly, Dugbe orthonairovirus (DUGV), while genetically and antigenically close to CCHFV, can produce false positives in certain CCHFV tests, particularly immunofluorescence assays (24). Therefore, CCHFV prevalence might be overestimated in areas where HAZV and DUGV are present. ELISAs are considered to have the highest specificity, followed by micro-virus neutralization tests (mVNT), indirect immunofluorescence assays (iIFA), and plaque reduction neutralization tests (PRNT) (25).

Virus neutralization assays, known for their high specificity, are rarely used for diagnosing CCHFV due to the requirement of high-containment laboratories (BSL-3/BSL-4) for handling live viruses. The level of containment depends on whether the area is endemic or non-endemic. Alternative methods, such as the pseudovirus neutralization test (pVNT), which uses pseudotyped Vesicular Stomatitis Virus, and the surrogate virus neutralization test (sVNT), can be performed in lower-containment BSL-2 laboratories, making them more accessible for diagnostic purposes (26, 27).

### 2.3 CCHFV in domestic animals

**Figure 3:**
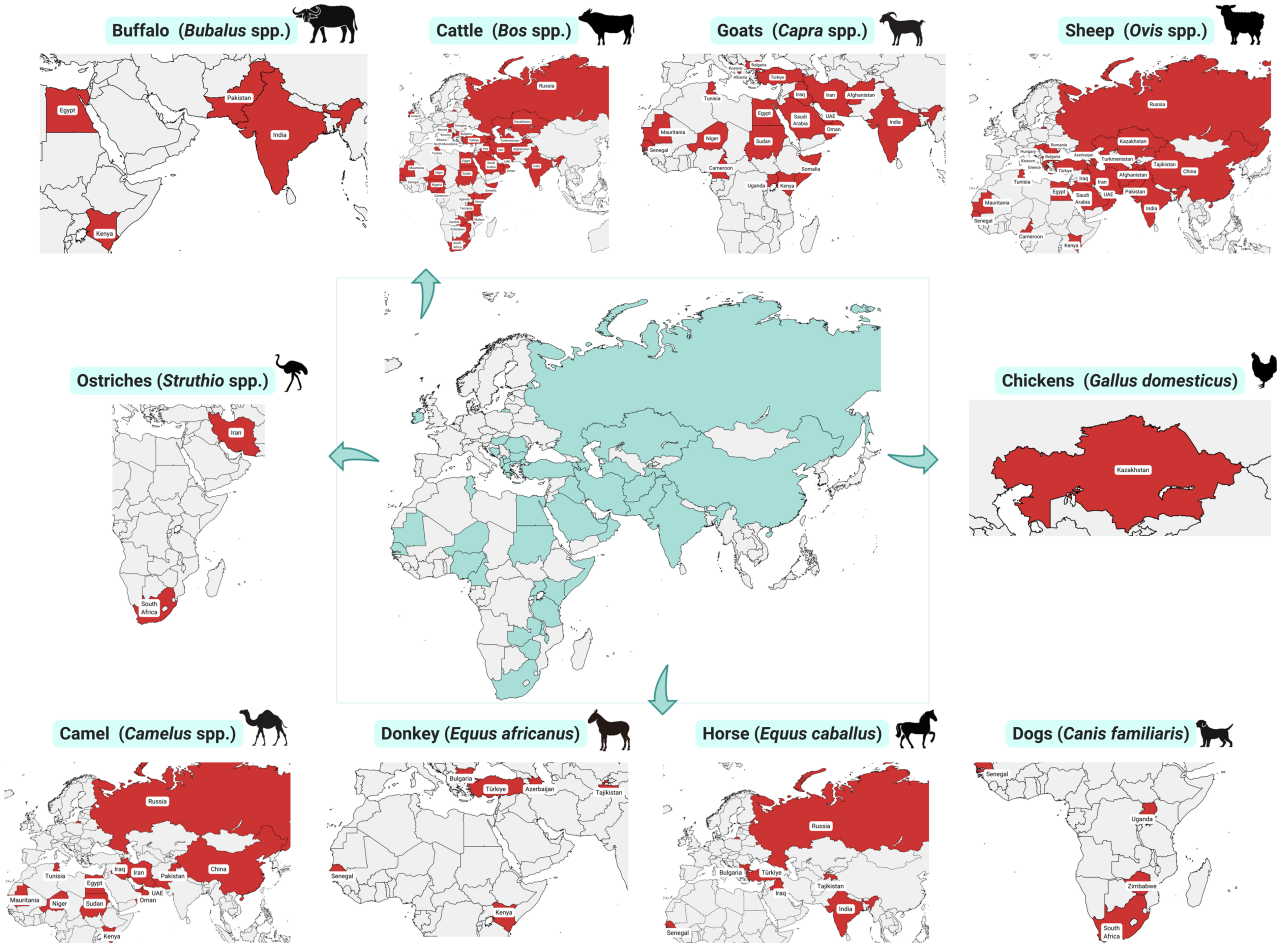
Geographic distribution of Crimean-Congo haemorrhagic fever virus exposure detected in domestic animals.

CCHFV circulates silently in an enzootic tick-vertebrate-tick cycle, without manifesting disease in animals. In humans, however, it triggers severe illness. Seroepidemiological surveys have identified CCHFV antibodies in various domestic animals (5) (Table 2; Figure 3). These surveys are crucial for identifying potential sources of CCHFV that might otherwise remain undetected. Since infected animals show no clinical symptoms, serological investigations are essential for assessing CCHFV exposure in animals and the associated risks for human exposure to infected ticksticks (4).

**Table 2:** List of seropositive domestic animals infected by Crimean-Congo haemorrhagic fever virus.

Artiodactyla	Bovidae	Buffalo	*Bubalus* spp.	Egypt, India, Kenya, Pakistan	(39, 41, 60, 164)
		Cattle	*Bos* spp.	Afghanistan, Albania, Armenia, Azerbaijan, Bosnia, Bulgaria, Cameroon, Egypt, Hungary, India, Iran, Iraq, Ireland, Kazakhstan, Kenya, Kosovo, Malawi, Mauritania, Niger, Nigeria, North Macedonia, Oman, Russia, Saudi Arabia, Senegal, Somalia, South Africa, Sudan, Tajikistan, Turkmenistan, Tanzania, Tunisia, Türkiye, United Arab Emirates, Uganda, Zambia, Zimbabwe	(12, 29–32, 39, 41–45, 47, 60, 61, 88, 108, 124, 145, 154, 164–200)
		Goats	*Capra* spp.	Afghanistan, Albania, Bulgaria, Cameroon, Egypt, India, Iran, Iraq, Kenya, Kosovo, Mauritania, Niger, Oman, Saudi Arabia, Senegal, Somalia, Sudan, Tunisia, Türkiye, United Arab Emirates, Uganda	(29, 39, 42–44, 61, 67, 108, 124, 139, 153, 154, 164, 172, 174–176, 178, 180, 186, 189, 190, 192–194, 197–203)
		Sheep	*Ovis* spp.	Afghanistan, Azerbaijan, Bulgaria, Cameroon, China, Egypt, Greece, Hungary, India, Iran, Iraq, Kazakhstan, Kenya, Kosovo, Mauritania, Oman, Pakistan, Romania, Russia, Saudi Arabia, Senegal, Tajikistan, Tunisia, Türkiye, Turkmenistan, United Arab Emirates	(4, 5, 12, 29, 42, 44, 47, 51, 124, 132, 153, 154, 162, 164, 167, 176, 178, 180, 186, 190–194, 196–200, 204–210)
	Camelidae	Camels	*Camelus* spp.	China, Egypt, Iran, Iraq, Kenya, Mauritania, Niger, Oman, Pakistan, Russia, Sudan, Tunisia, United Arab Emirates	(44, 124, 132, 138, 162, 173, 180, 186, 192, 193, 196, 211–216)
Perissodactyla	Equidae	Donkey	*Equus africanus*	Azerbaijan, Bulgaria, Kenya, Senegal, Tajikistan, Türkiye	(29, 42, 47, 48, 52, 167)
		Horses	*Equus caballus*	Bulgaria, India, Iraq, Russia, Senegal, Tajikistan, Türkiye	(42–45, 47, 48, 52, 182)
Carnivora	Canidae	Dogs	*Canis familiaris*	Senegal, South Africa, Uganda, Zimbabwe	(52–54)
Galliformes	Phasianidae	Chickens	*Gallus domesticus*	Kazakhstan	(177)
Struthioniformes	Struthionidae	Ostriches	*Struthio* spp.	Iran, South Africa	(56, 57)

These surveys, especially in CCHFV-endemic regions, reveal high levels of antibodies in cattle, sheep, goats, horses, camels, and other domestic animals, indicating their significant role in the epidemiology of CCHF. These animals support tick reproduction and facilitate the movement of ticks across large areas, aiding the spread of the virus. Large mammals serve as hosts for the virus during viremia, acting as intermediaries and amplifiers between ticks. Various vertebrate hosts, particularly large ungulates, can transiently increase infection opportunities, enabling the virus to spread to other ticks feeding on these hosts. They can also contribute to CCHFV spread through co-feeding transmission, where ticks acquire the virus from infected ticks nearby, even if the host animal is not viremic (13). The movement of livestock, which may harbor infected ticks, significantly influences the spread of the virus (13). When livestock travel long distances, they can unknowingly transport infected ticks, as these ticks feed for an extended period. Unregulated trade movements of domestic animals could greatly elevate the risk of introducing infected ticks to new areas (28).

The prevalence of CCHFV antibodies among livestock varies based on factors like age and breed, highlighting different levels of susceptibility and exposure. Older animals typically have higher antibody levels due to repeated exposure, while younger animals, such as calves, are more likely to contract the infection while grazing, increasing their chances of encountering infected ticks (29–32). Cross-bred cattle often show higher seropositivity compared to native breeds, possibly due to genetic or environmental factors L (32). Longitudinal studies suggest that animals with existing antibodies and tick infestations may be at risk of reinfection (19). Antibodies against CCHFV can persist in naturally infected livestock for up to 2 months, emphasizing the need for effective surveillance and control strategies (19).

The detection of CCHFV antibodies in domestic animals has been crucial in identifying the presence of the virus and localizing areas with higher risks of human infection. Livestock such as cattle, sheep, camels, and goats commonly become infected with CCHFV through tick bites, often experiencing asymptomatic transient viremia for 7–15 days (33, 34). Other domestic species, including buffaloes, horses, donkeys, dogs, chickens, and ostriches, occasionally show CCHFV seropositivity, though less commonly than livestock.

Buffaloes play an important role in CCHFV epidemiology as definitive hosts for *Hyalomma* and *Rhipicephalus* ticks. In a study examining the sera of 880 buffaloes, using ELISA, 145 were found to have been exposed to the virus (35). Their resistance to tick bites due to thicker hides and mud wallowing habits reduces the likelihood of tick-borne pathogen transmission (36–38). However, in densely populated regions like India, buffaloes may increase the risk of CCHFV transmission to humans (39, 40). In Africa, the coexistence of buffaloes and cattle within integrated wildlife-livestock ranching systems suggests a potential reservoir role for buffaloes in CCHFV transmission. A recent study in Kenya observed higher CCHFV prevalence in buffaloes compared to cattle, indicating that buffaloes could act as a reservoir, potentially transmitting the infection to cattle due to shared habitats and overlapping ranges (41).

Horses are susceptible to CCHFV infection and can serve as hosts for infected adult ticks, thereby contributing to virus transmission. They can produce antibody levels similar to other animals, but their viremia is too low to infect new naive ticks and sustain transmission through blood feeding (34). Seroprevalence studies have documented CCHFV prevalence in horses across various endemic regions, including Bulgaria (4, 42), India (43), Iraq (44), Russia (45, 46), Tajikistan (47), and Türkiye (48). The role of horses in CCHFV transmission varies depending on environmental conditions, tick prevalence, and the density of horse populations in endemic regions. In regions invaded by *H. marginatum* ticks such as the Czechia (49) and France (50), horses exhibit higher infestation rates compared to other ungulates, likely due to regular ectoparasite checks.

Donkeys play a crucial role in the spread of CCHFV as they frequently encounter ticks during rural activities. Along with mules, they have historically been vital in agriculture and transportation. The high seroprevalence of CCHFV in donkeys is influenced by factors such as climate, animal movement, living conditions, and cohabitation with other livestock, highlighting their role in sustaining the virus within communities. Although donkeys might not directly transmit the virus like viremic livestock, they significantly contribute to its persistence. Spengler et al. (5) reported seroprevalence rates of 18.8% in Azerbaijan, 17.4 and 50% in Bulgaria, and 39.5% in Tajikistan. In Kenya, Omoga et al. (51) found the highest seropositivity in donkeys at 31.4% compared to other livestock species. In Senegal, Mangombi et al. (52) reported a seropositivity rate of 17.2% in donkeys. The highest recorded seroprevalence was in Türkiye, where Saltik (48) reported a rate of 53.48% in donkeys.

Dogs can harbor CCHFV asymptomatically or with mild symptoms when exposed to infected ticks. Studies in Africa have shown varying seroprevalence rates among domestic dogs. Antibodies to CCHFV were found in 6% of dogs (*n* = 1978) in South Africa and Zimbabwe (53). In Senegal, Mangombi et al. (52) found a seropositivity rate of 18.2% in dogs. In Uganda, Atim et al. (54) reported a high seropositivity rate of 56.2% in dogs. While the role of dogs in CCHFV epidemiology is not as well understood as that of livestock, their close association with humans raises concerns about the potential introduction of infected ticks into human environments. Further research into companion animals and their interactions with vector species is essential to better understand their role in the ecology of CCHFV.

While various domestic mammals are susceptible to CCHFV infection, birds generally seem refractory. For example, Spengler et al. (34) stated a seroprevalence of 0.2% in chickens and ducks in Kazakhstan. Interestingly, ostriches demonstrate the presence of both CCHFV antibodies and viremia, unlike most other bird species. Ostriches appear to be the only birds in which there is detectable circulation of the virus in blood comparable to mammals (7). Viremia in ostriches is short, lasting 1–4 days, while the virus persists in visceral organs such as the spleen, liver, and kidneys for up to 5 days (55). Their role in transmitting the virus to humans is uncertain, but instances of notable viremia associated with CCHFV infection in humans have been noted (55–58).

### 2.4 CCHFV in wild animals

Numerous serological studies across a wide range of wild animals have highlighted the diverse responses observed in populations regarding CCHFV infections. These studies suggest their roles as amplifying hosts, facilitating virus transmission between infected and uninfected ticks during co-feeding or when feeding on a viremic animal.

**Figure 4:**
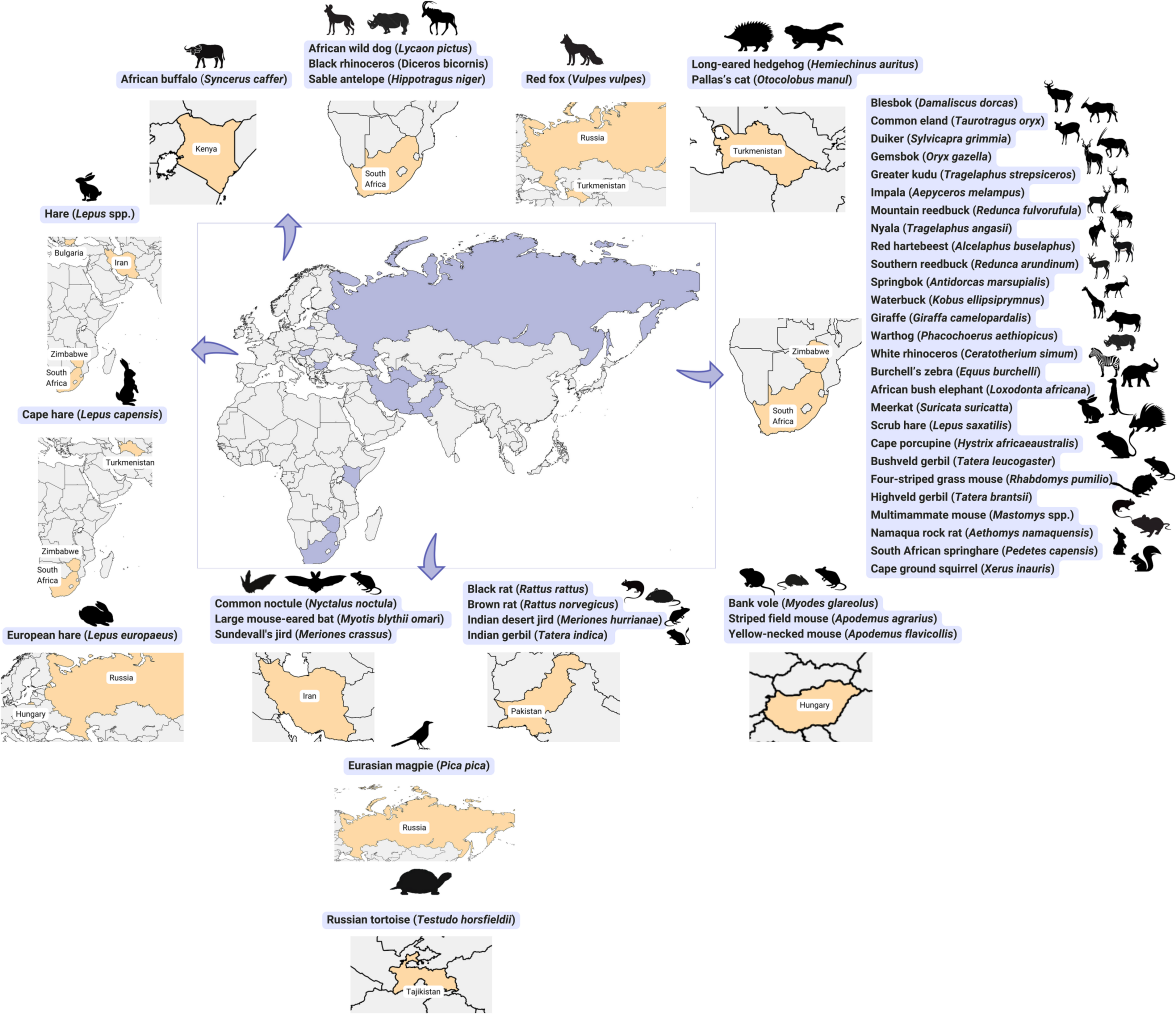
Geographic distribution of Crimean-Congo haemorrhagic fever virus exposure detected in wild animals.

A comprehensive review of nearly 7,000 samples from over 175 mammalian, avian, and reptilian species revealed varying levels of seroprevalence (Table 3; Figure 4) (5). Certain mammals, such as hares (3–22%), buffalo (10–75%), and rhinoceroses (40–68%), exhibited considerable seropositivity.

**Table 3:** List of seropositive wild animals infected by Crimean-Congo haemorrhagic fever virus.

Class	Order	Family	Common name	Scientific name	Country	References
Mammals	Artiodactyla	Bovidae	African buffalo	*Syncerus caffer*	Kenya	(41)
			Blesbok	*Damaliscus dorcas*	South Africa, Zimbabwe	(53)
			Common eland	*Taurotragus oryx*	South Africa, Zimbabwe	(53)
			Duiker	*Sylvicapra grimmia*	South Africa, Zimbabwe	(53)
			Gemsbok	*Oryx gazella*	South Africa, Zimbabwe	(53)
			Greater kudu	*Tragelaphus strepsiceros*	South Africa, Zimbabwe	(53, 62)
			Impala	*Aepyceros melampus*	South Africa, Zimbabwe	(53, 62)
			Mountain reedbuck	*Redunca fulvorufula*	South Africa, Zimbabwe	(53)
			Nyala	*Tragelaphus angasii*	South Africa, Zimbabwe	(53)
			Red hartebeest	*Alcelaphus buselaphus*	South Africa, Zimbabwe	(53)
			Sable antelope	*Hippotragus niger*	South Africa	(53, 62)
			Southern reedbuck	*Redunca arundinum*	South Africa, Zimbabwe	(53)
			Springbok	*Antidorcas marsupialis*	South Africa, Zimbabwe	(53)
			Waterbuck	*Kobus ellipsiprymnus*	South Africa, Zimbabwe	(53)
		Giraffidae	Giraffe	*Giraffa camelopardalis*	South Africa, Zimbabwe	(53, 62)
		Suidae	Warthog	*Phacochoerus aethiopicus*	South Africa, Zimbabwe	(53)
	Perissodactyla	Rhinocerotidae	White rhinoceros	*Ceratotherium simum*	South Africa, Zimbabwe	(53, 62)
			Black rhinoceros	*Diceros bicornis*	South Africa	(53, 62)
		Equidae	Burchell’s zebra	*Equus burchelli*	South Africa, Zimbabwe	(53)
	Proboscidea	Elephantidae	African bush elephant	*Loxodonta africana*	South Africa, Zimbabwe	(53)
	Carnivora	Canidae	African wild dog	*Lycaon pictus*	South Africa	(62)
			Red fox	*Vulpes vulpes*	Russia, Turkmenistan	(217, 218)
		Felidae	Pallas’s cat	*Otocolobus manul*	Turkmenistan	(218)
		Herpestidae	Meerkat	*Suricata suricatta*	South Africa, Zimbabwe	(53)
	Chiroptera	Vespertilionidae	Common noctule	*Nyctalus noctula*	Iran	(61)
			Large mouse-eared bat	*Myotis blythii omari*	Iran	(61)

	Lagomorpha	Leporidae	Cape hare	*Lepus capensis*	South Africa, Turkmenistan, Zimbabwe	(4, 53)
			Hare	*Lepus* spp.	Bulgaria, Iran, South Africa, Zimbabwe	(4, 42, 53, 219)
			European hare	*Lepus europaeus*	Russia, Hungary	(4, 217, 220)
			Scrub hare	*Lepus saxatilis*	South Africa, Zimbabwe	(53)
	Eulipotyphla	Erinaceidae	Long-eared hedgehog	*Hemiechinus auritus*	Turkmenistan	(4, 221)
		Cricetidae	Bank vole	*Myodes glareolus*	Hungary	(222)
	Rodentia	Hystricidae	Cape porcupine	*Hystrix africaeaustralis*	South Africa, Zimbabwe	(53)
		Muridae	Black rat	*Rattus rattus*	Pakistan	(60)
		Muridae	Brown rat	*Rattus norvegicus*	Pakistan	(60)
		Muridae	Bushveld gerbil	*Gerbilliscus leucogaster*	South Africa, Zimbabwe	(53)
		Muridae	Four-striped grass mouse	*Rhabdomys pumilio*	South Africa, Zimbabwe	(53)
		Muridae	Highveld gerbil	*Tatera brantsii*	South Africa, Zimbabwe	(53)
		Muridae	Indian desert jird	*Meriones hurrianae*	Pakistan	(60)
		Muridae	Indian gerbil	*Tatera indica*	Pakistan	(60)
		Muridae	Multimammate mouse	*Mastomys* spp. *(coucha, natalensis)*	South Africa, Zimbabwe	(53)
		Muridae	Namaqua rock rat	*Aethomys namaquensis*	South Africa, Zimbabwe	(53)
		Muridae	Striped field mouse	*Apodemus agrarius*	Hungary	(222)
		Muridae	Sundevall’s jird	*Meriones crassus*	Iran	(61)
		Muridae	Yellow-necked mouse	*Apodemus flavicollis*	Hungary	(222)
		Pedetidae	South African springhare	*Pedetes capensis*	South Africa, Zimbabwe	(53)
Sciuridae		Cape ground squirrel	*Xerus inauris*	South Africa, Zimbabwe	(53)
Aves	Passeriformes	Corvidae	Eurasian magpie	*Pica pica*	Russia	(217)
Reptilia	Testudines	Testudinidae	Russian tortoise	*Testudo horsfieldii*	Tajikistan	(223)

Rodents and lagomorphs are crucial in CCHFV epidemiology (4, 7, 59). Several rodent and lagomorph species, including the European hare (*Lepus europaeus*), scrub hare (*Lepus saxatilis*), Cape hare (*Lepus capensis*), bushveld gerbil (*Gerbilliscus leucogaster*), four-striped grass mouse (*Rhabdomys pumilio*), and multimammate mouse (*Mastomys* spp.), act as amplifying hosts, facilitating virus replication and transmission to ticks during their feeding (7). Infected rodents contribute significantly to the spread of CCHFV by transmitting the virus to ticks, thereby influencing its presence in the environment. Understanding the role of rodents in CCHFV transmission is important for developing effective surveillance and control strategies. Various rodent species such as the Cape porcupine (*Hystrix africaeaustralis*) (53), black rat (*Rattus rattus*) (60), brown rat (*R. norvegicus*) (60), bushveld gerbil (*G. leucogaster*) (53), four-striped grass mouse (*R. pumilio*) (53), Highveld gerbil (*Tatera brantsii*) (53), Indian desert jird (*Meriones hurrianae*) (60), Indian gerbil (*T. indica*) (60), multimammate mouse (*Mastomys* spp.) (53), Namaqua rock rat (*Aethomys namaquensis*) (53), Sundevall’s jird (*M. crassus*) (61), South African springhare (*Pedetes capensis*) (53), and Cape ground squirrel (*Xerus inauris*) (53) have displayed seropositivity to CCHFV in different regions, indicating their potential involvement in the virus’s transmission cycle.

Additionally, other animals, including many large herbivorous mammals within the Artiodactyla and Perissodactyla orders, such as the African buffalo (*Syncerus caffer*), blesbok (*Damaliscus dorcas*) (53), common eland (*Taurotragus oryx*) (53), duiker (*Sylvicapra grimmia*) (53), gemsbok (*Oryx gazella*) (53), greater kudu (*Tragelaphus strepsiceros*) (53, 62), impala (*Aepyceros melampus*) (53, 62), mountain reedbuck (*Redunca fulvorufula*) (53), nyala (*Tragelaphus angasii*) (53, 62), red hartebeest (*Alcelaphus buselaphus*) (53), sable antelope (*Hippotragus niger*), southern reedbuck (*Redunca arundinum*) (53), springbok (*Antidorcas marsupialis*) (53), waterbuck (*Kobus ellipsiprymnus*) (53), giraffe (*Giraffa camelopardalis*) (53), warthog (*Phacochoerus aethiopicus*) (53), white rhinoceros (*Ceratotherium simum*) (53, 62), black rhinoceros (*Diceros bicornis*) (53, 62), and Burchell’s zebra (*Equus burchelli*) (53), as well as the African bush elephant (*Loxodonta africana*) (53, 62) in South Africa and Zimbabwe, have demonstrated seropositivity to CCHFV.

Certain members of the Carnivora order also exhibited seropositivity in specific regions, including the African wild dog (*Lycaon pictus*) (62) in South Africa, red fox (*Vulpes vulpes*) in Russia and Turkmenistan (4), and Pallas’s cat (*Otocolobus manul*) in Turkmenistan (4).

Bats, such as the common noctule (*Nyctalus noctula*) and large mouse-eared bat (*Myotis blythii omari*) in Iran (61), also displayed seropositivity to CCHFV.

The potential involvement of birds in transmitting and maintaining CCHFV poses a significant concern in disease ecology. Migratory birds, traveling long distances through various habitats, carry a range of ectoparasites like ticks, mites, fleas, and lice. Their movements, especially between Africa and Europe, coincide with environmental changes that may affect the spread of tick-borne diseases. Studies show migratory birds can transport *H. marginatum* ticks from Africa to Europe, with certain Passerine bird species (e.g., *Acrocephalus arundinaceus*, *A. scirpaceus*, *A. palustris*, *A. schoenobaenus*, *Locustella luscinioides*, and *Luscinia megarhynchos*) facilitating the dispersion of infected ticks along their migratory routes (59). Although avian species may be refractory to CCHFV infection (5, 34, 56, 63), they can serve as blood sources for immature *H. marginatum* ticks, potentially contributing to disease spread. While most wild birds do not show evidence of CCHFV infection, exceptions like magpies (*Pica pica*), which have displayed CCHFV antibodies, suggest a more complex situation (7). Ostriches, however, show unique susceptibility to CCHFV, displaying both antibodies and viremia, unlike other birds (7). Further research is crucial to understand how different bird species contribute to CCHFV transmission.

Among reptiles, only one species—the Horsfield’s tortoise (*Testudo horsfieldii*) in Tajikistan—has been reported as seropositive for CCHFV (4). Notably, the tick species *H. aegyptium*, which is closely associated with tortoises and often linked to CCHFV transmission (64, 70), primarily infests hosts within the *Testudo* genus. This suggests a possible role of tortoises in virus transmission. However, the overall susceptibility of reptiles to CCHFV remains unclear, despite evidence pointing to potential transmission through tortoise-associated ticks.

### 2.5 Molecular detection of CCHFV in animals

Despite evidence of seropositivity among domestic and wild animals, isolating CCHFV directly from these hosts has proven challenging and direct CCHFV isolation from animals is scarce (5). Documented instances of direct CCHFV isolation from animals remain scarce, with notable cases including a febrile cow in Kenya (43), cattle and a goat from a Nigerian abattoir (90), a sentinel goat in Senegal (4, 43), European hares in Crimea (67), and a hedgehog in Nigeria (90). These sporadic cases highlight the difficulties in identifying and isolating the virus due to the typically short viremic period in infected animals and the absence or mildness of clinical symptoms (7). As a result, most successful isolations come from ticks or human cases, where the virus is more prominent.

Molecular detection of CCHFV infection relies on both real-time and end-point PCR techniques (68). These methods amplify specific segments of the viral RNA, such as the S segment encoding the nucleoprotein, enabling precise detection and quantification of the virus. In resource-limited settings, loop-mediated isothermal amplification (RT-LAMP) offers a cost-effective alternative, amplifying viral RNA under isothermal conditions without the need for advanced equipment (69).

Enhanced molecular detection methods, longitudinal studies, and comprehensive monitoring programs are essential for fully understanding the role of various animal species in the ecology of CCHFV. This knowledge is important for mitigating potential transmission risks to humans and preventing outbreaks of this serious zoonotic disease.

### 2.6 Experimental CCHFV infections in animals

Experimental studies investigating CCHFV infections across various animal species have provided valuable insights into susceptibility patterns, infection dynamics, and immune responses.

Small mammals, despite displaying short viremic periods of 2 to 15 days followed by antibody development, are not considered long-term reservoirs for CCHFV (34). Nonetheless, population surges in species like hares have been linked to disease outbreaks, implying their ecological significance in CCHFV transmission (7, 34). Studies on small African wild mammals and laboratory animals showed diverse responses to CCHFV, with some species showing viremia and antibody responses, while others did not. South African hedgehogs, for instance, display resistance but develop neutralizing antibodies (71). Furthermore, the virus was recovered from the blood of experimentally infected long-eared hedgehogs (*Hemiechinus auritus*) (4), while European hedgehogs (*Erinaceus europaeus*) did not exhibit similar susceptibility (72). The varying outcomes among hedgehog species indicate that susceptibility to CCHFV and infection dynamics may vary even within closely related species.

Experimental studies have shown that various rodent and lagomorph species respond differently to CCHFV infection. European hares (*Lepus europaeus*), for example, showed varying viremic intervals (2, 4, 5, 9 dpi) and generated an antibody response by day 7, which was maintained throughout the study (34). Similarly, scrub hares (*Lepus saxatilis*) and bushveld gerbils (*G. leucogaster*) exhibited viremia within the first week after infection, along with the production of antibodies (71). However, some species like the Cape ground squirrel (*Xerus inauris*) and the four-striped grass mouse (*Rhabdomys pumilio*) showed limited or no viremia and inconsistent antibody responses (71). On the other hand, the Southern multimammate mouse (*Mastomys coucha*), white-tailed rat (*Uromys caudimaculatus*), and red veld rat (*Aethomys chrysophilus*) demonstrated viremia (ranging from 1 to 6 dpi) and produced antibodies, indicating different responses to CCHFV among rodent species (71). Guinea pigs displayed low-level viremia accompanied by elevated temperatures. The onset of viremia correlated with the route of infection (71). The varied responses among small mammals highlight the complexity of CCHFV interactions, emphasizing the need for species-specific understanding in ecological dynamics. For a more comprehensive list, we encourage referring to the detailed experimental infection data of various small mammals infected with CCHFV, as thoroughly discussed in these studies (7, 34).

Experimental studies have investigated how CCHFV infects livestock, focusing on ruminants like sheep, cattle, horses and donkeys. Similar to small mammals, these ruminants experienced a brief period of viremia and developed antibodies about a week after inoculation (34). In sheep, maternal transfer of these antibodies was demonstrated, indicating a form of passive immunity (73). Additionally, experiments on West African sheep highlighted diverse clinical manifestations following infection (73). Some infected sheep developed moderate fever, hepatic dysfunction, and abnormal blood cell counts, including marked neutrophilia, that persisted for weeks. These observations highlight the potential impacts of CCHFV infection in livestock, particularly in sheep, affecting their health and possibly contributing to the virus’s circulation in nature. Calves have also been subjects of experimental infections, showing varying responses based on their age at the time of infection (74). When infected, 2-month-old calves displayed mild illness, with the virus detected in their blood. In contrast, 6-month-old calves did not show signs of viremia. However, only the younger calves, with detectable viremia, would be significant for the virus’s circulation, despite both age groups exhibiting high levels of antibodies against CCHFV. Horses and donkeys showed different responses: donkeys exhibited low-level viremia (75), while horses displayed minimal or no viremia but developed strong virus-neutralizing antibodies for up to 3 months (76). This highlights horses as valuable sources of serum for diagnostic and therapeutic purposes due to the stability of their virus-neutralizing antibodies.

It is important to note that these experimental studies were conducted in the 1970s. These studies revealed low viremia levels and asymptomatic cases in many animals, yet some could still transmit the virus to ticks during feeding. These results emphasize the need for updated research to better understand current CCHFV dynamics in livestock and improve prevention strategies. On the other hand, performing such research would be very complicated or even impossible nowadays as CCHFV is classified as BSL-4 pathogen.

Efforts to establish animal models for CCHF have faced challenges, with limited success achieved so far. Newborn mice are the only animals besides humans that display symptoms of the disease, providing a basis for research. (7). Additionally, genetically modified adult mice and hamsters, deficient in specific immune components, mimic human disease and exhibit uncontrolled viral replication, inflammatory immune reactions, liver pathology, and mortality (77–81). Non-human primate models, such as cynomolgus macaques, reflect varied disease outcomes similar to humans, aiding in preclinical assessments of therapeutics and vaccines (82). In experimental infections with African green monkeys (*Chlorocebus sabaeus*), the majority of subjects exhibited either limited symptoms or remained asymptomatic, although one monkey developed fever post-infection, with some monkeys showing detectable antibodies against the virus (83). In a separate study, Patas monkeys (*Erythrocebus patas*) and a Guinea baboon (*Papio papio*) displayed low-level viremia following inoculation, ultimately leading to the development of neutralizing antibodies in the baboon (84).

Studies investigating CCHFV infection in birds suggest that avian species, both wild and domestic, are generally refractory to the virus. Early experiments found that birds remained healthy after CCHFV inoculation, displaying no signs of viremia or detectable antibody responses (87). However, several studies showed that ground-feeding birds may therefore contribute to the virus’s ecological dynamics by facilitating viremic, non-viremic transmission or cofeeding (7, 56, 85, 87). Ostriches, however, appear to be significant hosts for CCHFV, showing detectable viremia and epidemiologically linked to human infections (85). In controlled experiments, infected ostriches developed viremia and subsequently produced antibodies against CCHFV (88). Other bird species, for example the red-billed hornbill (*Tockus erythrorhynchus*), demonstrated replication of CCHFV without viremia but were able to infect immature naive ticks (85). Other birds, for example helmeted guineafowl (*Numida meleagris*), exhibited low-level viremia followed by a transient antibody response starting 5–6 dpi (56). Additionally, birds like the glossy starling (*Lamprotornis* spp.), did not display viremia but generated an antibody response (85). Further research is needed to clarify the role of birds in CCHFV transmission and its ecological implications.

## 3 Prevention and control of CCHFV in animals

Preventing and controlling the transmission of CCHFV in animals is crucial not only for animal health but also for preventing the virus from spreading to humans, where it poses a significant health risk. These measures aim to minimize the risk of virus transmission to humans and prevent CCHFV from reaching non-endemic regions.

The primary strategy to control CCHFV in animals involves managing tick populations, the main vectors for the virus. Using acaricides and other tick control methods is the most practical approach, although complete prevention of tick bites is unlikely (86). Efforts often focus on periods surrounding slaughter, when exposure of slaughterhouse workers to CCHFV in animal blood or tissues is most likely. Additional practices to reduce tick exposure include environmental adjustments, treating animals with tick repellents, maintaining clean pastures, establishing quarantine measures for new animals, and improving animal housing (86). Preventing or controlling CCHF infection in animals and ticks is complex due to the typically unnoticed tick-animal-tick-CCHFV life cycle and the often asymptomatic nature of the infection in most animals. The widespread presence of tick vectors further complicates control efforts, making acaricide-based tick control feasible only in well-managed livestock facilities.

Surveillance systems play a crucial role in early detection and response to CCHFV outbreaks in animals. Regular monitoring of animal populations in endemic areas for the presence of CCHFV antibodies or viral RNA could help identify potential reservoirs and understand disease dynamics. Timely detection enables prompt interventions to prevent further spread.

Finally, implementing biosecurity measures in farms, slaughterhouses, and veterinary facilities is essential to prevent CCHFV transmission between animals and humans as these facilities have been identified as major risk areas for human infection (8, 90, 224, 225).

Control strategies for CCHF infection in animals also extend to human protection. These strategies include avoiding tick bites through the use of repellents and employing adequate protection when handling or slaughtering animals (226). Preventing the movement of naive animals into endemic areas is crucial, as it minimizes the risk of vertebrate amplification of the virus, reducing occupational risks for workers involved in animal processing. Educating livestock owners, veterinarians, and the general public about CCHFV transmission, symptoms in animals, and preventive measures is vital. Raising awareness about the disease’s impact, emphasizing the importance of early reporting of suspected cases, and promoting proper biosecurity measures are key components of effective disease control efforts.

## 4 Conclusion

CCHFV, a highly virulent virus transmitted by *Hyalomma* ticks, poses a significant global health threat by causing severe haemorrhagic fever in humans. Its widespread presence across Africa, Asia, and Europe highlights the urgent need to understand its behavior within tick vectors and animal hosts.

Both wild and domestic animals, acting as asymptomatic carriers, play critical roles in maintaining tick populations and transmitting the virus, thereby potentially spreading the disease. Further, small mammals like hares and hedgehogs support immature tick populations, while larger domestic animals such as cattle, goats, and sheep can inadvertently expose humans to CCHFV during handling and slaughter. The complex interplay between the virus, ticks, and vertebrate hosts presents significant challenges in controlling CCHFV transmission. Despite often lacking visible symptoms, animals play a crucial role in the maintenance and spread of the virus, highlighting the necessity for rigorous surveillance, serological screening, and a deeper understanding of their roles in CCHFV ecology. Experimental infections confirm that various animal species are susceptible to CCHFV, emphasizing the need for ongoing research and monitoring.

Control strategies mainly focus on managing tick populations through the use of acaricides and improving hygiene in animal habitats. However, the virus’s elusive nature within animals and the difficulties in identifying infected hosts continue to pose significant challenges to disease control. Continued research and a deeper understanding of CCHFV in animal populations are essential for developing more effective control strategies, mitigating zoonotic risks, and protecting the health of both animals and humans.

## References

[1] Bente DA Forrester NL Watts DM McAuley AJ Whitehouse CA Bray M. Crimean-Congo hemorrhagic fever: history, epidemiology, pathogenesis, clinical syndrome and genetic diversity. Antivir Res. (2013) 100:159–89. doi: 10.1016/j.antiviral.2013.07.006, PMID: 23906741

[2] Mertens M Schmidt K Ozkul A Groschup MH. The impact of Crimean-Congo hemorrhagic fever virus on public health. Antivir Res. (2013) 98:248–60. doi: 10.1016/j.antiviral.2013.02.007, PMID: 23458713

[3] Shayan S Bokaean M Shahrivar MR Chinikar S. Crimean-Congo hemorrhagic fever. Lab Med. (2015) 46:180–9. doi: 10.1309/LMN1P2FRZ7BKZSCO, PMID: 26199256

[4] Hoogstraal H. The epidemiology of tick-borne Crimean-Congo hemorrhagic fever in Asia, Europe, and Africa. J Med Entomol. (1979) 15:307–417. doi: 10.1093/jmedent/15.4.307, PMID: 113533

[5] Spengler JR Bergeron É Rollin PE. Seroepidemiological studies of Crimean-Congo hemorrhagic fever virus in domestic and wild animals. PLoS Negl Trop Dis. (2016) 10:e0004210. doi: 10.1371/journal.pntd.0004210, PMID: 26741652 PMC4704823

[6] Hoch T Breton E Josse M Deniz A Guven E Vatansever Z. Identifying main drivers and testing control strategies for CCHFV spread. Exp Appl Acarol. (2016) 68:347–59. doi: 10.1007/s10493-015-9937-9, PMID: 26174420

[7] Ergönül Ö. Crimean-Congo haemorrhagic fever. Lancet Infect Dis. (2006) 6:203–14. doi: 10.1016/S1473-3099(06)70435-2, PMID: 16554245 PMC7185836

[8] Shahhosseini N Azari-Garmjan GA Khadem Rezaiyan M Haeri A Nowotny N Fooks AR. Factors affecting transmission of Crimean – Congo hemorrhagic fever among slaughterhouse employees: a Serosurvey in Mashhad, Iran. Jundishapur J Microbiol. (2018) 11:e57980. doi: 10.5812/jjm.57980

[9] Sargianou M Panos G Tsatsaris A Gogos C Papa A. Crimean-Congo hemorrhagic fever: seroprevalence and risk factors among humans in Achaia, western Greece. Int J Infect Dis. (2013) 17:e1160–5. doi: 10.1016/j.ijid.2013.07.015, PMID: 24084247

[10] Sharifi-Mood B Metanat M Alavi-Naini R. Prevalence of Crimean-Congo hemorrhagic FeverAmong high risk human groups. Int J High Risk Behav Addict. (2014) 3:e11520. doi: 10.5812/ijhrba.11520, PMID: 24971294 PMC4070186

[11] Chinikar S Ghiasi SM Moradi M Goya MM Shirzadi MR Zeinali M. Geographical distribution and surveillance of Crimean-Congo hemorrhagic fever in Iran. Vector Borne Zoonotic Dis. (2010) 10:705–8. doi: 10.1089/vbz.2009.0247, PMID: 20854025

[12] Mustafa ML Ayazi E Mohareb E Yingst S Zayed A Rossi CA. Crimean-Congo hemorrhagic fever, Afghanistan, 2009. Emerg Infect Dis. (2011) 17:1940–1. doi: 10.3201/eid1710.110061, PMID: 22000377 PMC3310665

[13] Gargili A Estrada-Peña A Spengler JR Lukashev A Nuttall PA Bente DA. The role of ticks in the maintenance and transmission of Crimean-Congo hemorrhagic fever virus: a review of published field and laboratory studies. Antivir Res. (2017) 144:93–119. doi: 10.1016/j.antiviral.2017.05.010, PMID: 28579441 PMC6047067

[14] Gray JS Dautel H Estrada-Peña A Kahl O Lindgren E. Effects of climate change on ticks and tick-borne diseases in Europe. Interdiscip Perspect Infect Dis. (2009) 2009:1–12. doi: 10.1155/2009/593232, PMID: 19277106 PMC2648658

[15] Celina SS Černý J Samy AM. Mapping the potential distribution of the principal vector of Crimean-Congo haemorrhagic fever virus *Hyalomma marginatum* in the Old World. PLoS Negl Trop Dis. (2023) 17:e0010855. doi: 10.1371/journal.pntd.0010855, PMID: 38011221 PMC10703407

[16] Nasirian H. Ticks infected with Crimean-Congo hemorrhagic fever virus (CCHFV): a decision approach systematic review and meta-analysis regarding their role as vectors. Travel Med Infect Dis. (2022) 47:102309. doi: 10.1016/j.tmaid.2022.102309, PMID: 35318129

[17] Petrova ID Kononova YV Chausov EV Shestopalov AM Tishkova FH. Genetic variants of the Crimean-Congo hemorrhagic fever virus circulating in endemic areas of southern Tajikistan in 2009. Mol Genet Microbiol Virol. (2013) 28:119–26. doi: 10.3103/S0891416813030063 24364143

[18] Albayrak H Ozan E Kurt M. An antigenic investigation of Crimean-Congo hemorrhagic fever virus (CCHFV) in hard ticks from provinces in northern Turkey. Trop Anim Health Prod. (2010) 42:1323–5. doi: 10.1007/s11250-010-9579-1, PMID: 20401757

[19] Zeller HG Cornet J-P Diop A Camicas J-L. Crimean—Congo hemorrhagic fever in ticks (Acari: Ixodidae) and ruminants: field observations of an epizootic in Bandia, Senegal (1989–1992). J Med Entomol. (1997) 34:511–6. doi: 10.1093/jmedent/34.5.511, PMID: 9379454

[20] Gergova I Kunchev M Kamarinchev B. Crimean-Congo hemorrhagic fever virus-tick survey in endemic areas in Bulgaria. J Med Virol. (2012) 84:608–14. doi: 10.1002/jmv.23214, PMID: 22337300

[21] Mazzola LT Kelly-Cirino C. Diagnostic tests for Crimean-Congo haemorrhagic fever: a widespread tickborne disease. BMJ Glob Health. (2019) 4:e001114. doi: 10.1136/bmjgh-2018-001114, PMID: 30899574 PMC6407549

[22] Gülce-İz S Elaldı N Can H Şahar EA Karakavuk M Gül A. Development of a novel recombinant ELISA for the detection of Crimean-Congo hemorrhagic fever virus IgG antibodies. Sci Rep. (2021) 11:5936. doi: 10.1038/s41598-021-85323-1, PMID: 33723328 PMC7961021

[23] Hartlaub J von Arnim F Fast C Somova M Mirazimi A Groschup MH. Sheep and cattle are not susceptible to experimental inoculation with Hazara Orthonairovirus, a tick-borne arbovirus closely related to CCHFV. Microorganisms. (2020) 8:1927. doi: 10.3390/microorganisms8121927, PMID: 33291703 PMC7761912

[24] Hartlaub J Daodu OB Sadeghi B Keller M Olopade J Oluwayelu D. Cross-reaction or co-infection? Serological discrimination of antibodies directed against Dugbe and Crimean-Congo hemorrhagic fever Orthonairovirus in Nigerian cattle. Viruses. (2021) 13:1398. doi: 10.3390/v13071398, PMID: 34372604 PMC8310240

[25] Hartlaub J Keller M Groschup MH. Deciphering antibody responses to Orthonairoviruses in ruminants. Microorganisms. (2021) 9:1493. doi: 10.3390/microorganisms9071493, PMID: 34361926 PMC8303759

[26] Wang Q Wang S Shi Z Li Z Zhao Y Feng N. Establishment of two serological methods for detecting IgG and neutralizing antibodies against Crimean-Congo hemorrhagic fever virus glycoprotein. Front Cell Infect Microbiol. (2024) 14:14. doi: 10.3389/fcimb.2024.1341332, PMID: 38746783 PMC11091404

[27] Suda Y Chamberlain J Dowall SD Saijo M Horimoto T Hewson R. The development of a novel diagnostic assay that utilizes a Pseudotyped vesicular stomatitis virus for the detection of neutralizing activity against Crimean-Congo hemorrhagic fever virus. Jpn J Infect Dis. (2018) 71:205–8. doi: 10.7883/yoken.JJID.2017.354, PMID: 29709967

[28] Alam MM Khurshid A Sharif S Shaukat S Rana MS Angez M. Genetic analysis and epidemiology of Crimean Congo hemorrhagic fever viruses in Baluchistan province of Pakistan. BMC Infect Dis. (2013) 13:201. doi: 10.1186/1471-2334-13-201, PMID: 23641865 PMC3652740

[29] Barthel R Mohareb E Younan R Gladnishka T Kalvatchev N Moemen A. Seroprevalance of Crimean-Congo haemorrhagic fever in Bulgarian livestock. Biotechnol Biotechnol Equip. (2014) 28:540–2. doi: 10.1080/13102818.2014.931685, PMID: 26019541 PMC4434116

[30] Lotfollahzadeh S Nikbakht Boroujeni GR Mokhber Dezfouli MR Bokaei S. A Serosurvey of Crimean-Congo Haemorrhagic fever virus in dairy cattle in Iran. Zoonoses Public Health. (2011) 58:54–9. doi: 10.1111/j.1863-2378.2009.01269.x, PMID: 19912604

[31] Ibrahim AM Adam IA Osman BT Aradaib IE. Epidemiological survey of Crimean Congo hemorrhagic fever virus in cattle in East Darfur state, Sudan. Ticks Tick Borne Dis. (2015) 6:439–44. doi: 10.1016/j.ttbdis.2015.03.002, PMID: 25898993

[32] Adam IA Mahmoud MAM Aradaib IE. A seroepidemiological survey of Crimean Congo hemorrhagic fever among cattle in North Kordufan state, Sudan. Virol J. (2013) 10:178. doi: 10.1186/1743-422X-10-178, PMID: 23738961 PMC3679941

[33] Spengler JR Bergeron É Spiropoulou CF. Crimean-Congo hemorrhagic fever and expansion from endemic regions. Curr Opin Virol. (2019) 34:70–8. doi: 10.1016/j.coviro.2018.12.002, PMID: 30660091 PMC6497153

[34] Spengler JR Estrada-Peña A Garrison AR Schmaljohn C Spiropoulou CF Bergeron É. A chronological review of experimental infection studies of the role of wild animals and livestock in the maintenance and transmission of Crimean-Congo hemorrhagic fever virus. Antivir Res. (2016) 135:31–47. doi: 10.1016/j.antiviral.2016.09.013, PMID: 27713073 PMC5102700

[35] El-Alfy E-S Abbas I Elseadawy R Saleh S Elmishmishy B El-Sayed SAE-S. Global prevalence and species diversity of tick-borne pathogens in buffaloes worldwide: a systematic review and meta-analysis. Parasit Vectors. (2023) 16:115. doi: 10.1186/s13071-023-05727-y, PMID: 36998029 PMC10061416

[36] Aubry P Geale DW. A review of bovine Anaplasmosis. Transbound Emerg Dis. (2011) 58:1–30. doi: 10.1111/j.1865-1682.2010.01173.x, PMID: 21040509

[37] Romero-Salas D Mira A Mosqueda J García-Vázquez Z Hidalgo-Ruiz M Vela NAO. Molecular and serological detection of Babesia bovis- and Babesia bigemina-infection in bovines and water buffaloes raised jointly in an endemic field. Vet Parasitol. (2016) 217:101–7. doi: 10.1016/j.vetpar.2015.12.030, PMID: 26827869

[38] Woolhouse MEJ Webster JP Domingo E Charlesworth B Levin BR. Biological and biomedical implications of the co-evolution of pathogens and their hosts. Nat Genet. (2002) 32:569–77. doi: 10.1038/ng1202-569, PMID: 12457190

[39] Mourya DT Yadav PD Shete AM Gurav YK Raut CG Jadi RS. Detection, isolation and confirmation of Crimean-Congo hemorrhagic fever virus in human, ticks and animals in Ahmadabad, India, 2010–2011. PLoS Negl Trop Dis. (2012) 6:e1653. doi: 10.1371/journal.pntd.0001653, PMID: 22616022 PMC3352827

[40] Sarangi L Mulpuri H Rana S Prasad A Muthappa P. Seroprevalence of Crimean-Congo haemorrhagic fever in Indian cattle and buffaloes. J Vector Borne Dis. (2023) 60:259–64. doi: 10.4103/0972-9062.364722, PMID: 37843236

[41] Obanda V Agwanda B Blanco-Penedo I Mwangi IA King’ori E Omondi GP. Livestock presence influences the Seroprevalence of Crimean Congo hemorrhagic fever virus on sympatric wildlife in Kenya. Vector Borne Zoonotic Dis. (2021) 21:809–16. doi: 10.1089/vbz.2021.0024, PMID: 34559011

[42] Vasilenko S Katsarov G Mikhailov A Teckharova M Levi V Levi S. Crimean hemorrhagic fever (CHF) in Bulgaria. Tr Inst Polio Virus Entsef. (1971) 19:100–11.

[43] Shanmugam J Smirnova SE Chumakov MP. Presence of antibody to arboviruses of the Crimean Haemorrhagic fever-Congo (CHF-Congo) group in human beings and domestic animals in India. Indian J Med Res. (1976) 64:1403–13. PMID: 828146

[44] Tantawi HH Shony MO Al-Tikriti SK. Antibodies to Crimean-Congo haemorrhagic fever virus in domestic animals in Iraq: a seroepidemiological survey. Int J Zoonoses. (1981) 8:115–20. Available at: http://europepmc.org/abstract/MED/6806205. PMID: 6806205

[45] Badalov M Butenko A Karinskaya G Leshchinskaya E Rubin S Tkachenko E. Results of serological investigation of the rural population and domestic animals in Rostov oblast in connection with the problem of prevention. (In English: NAMRU-T834). Mater 16 Nauch Sess Inst Polio Virus Entsef. (1969) 2:117–118.

[46] Berezin V Chumakov M Rubin S Stolbov D Butenko A Bashkirtsev V. Contribution to the ecology of Crimean hemorrhagic fever virus in the lower Volga River (NAMRU-T836). Arboviruses. (1969) 2:120–2.

[47] Smirnova SE Daniyarov OA Zgurskaya GN Kasymov KT Pavlovich AN Pak TP. Serological investigation of humans and animals in Tadzhik SSR for antibodies to Crimean hemorrhagic fever virus (from the 1968 data). (In English: NAMRU-T964). Tr Inst Polio Virus Entsef Akad Med Nauk SSSR. (1971) 19:66–71.

[48] Saltık HS. Tek tırnaklı hayvanlarda Kırım Kongo Hemorajik Ateşi Virusu’na spesifik antikorların tespiti. Kocatepe Vet J. (2022) 15:443–9. doi: 10.30607/kvj.1172589

[49] Lesiczka PM Daněk O Modrý D Hrazdilová K Votýpka J Zurek L. A new report of adult Hyalomma marginatum and *Hyalomma rufipes* in the Czech Republic. Ticks Tick Borne Dis. (2022) 13:101894. doi: 10.1016/j.ttbdis.2021.101894, PMID: 34996002

[50] Grech-Angelini S Stachurski F Lancelot R Boissier J Allienne J-F Marco S. Ticks (Acari: Ixodidae) infesting cattle and some other domestic and wild hosts on the French Mediterranean island of Corsica. Parasit Vectors. (2016) 9:582. doi: 10.1186/s13071-016-1876-8, PMID: 27842608 PMC5109666

[51] Omoga DCA Tchouassi DP Venter M Ogola EO Osalla J Kopp A. Transmission dynamics of Crimean–Congo Haemorrhagic fever virus (CCHFV): evidence of circulation in humans, livestock, and rodents in diverse ecologies in Kenya. Viruses. (2023) 15:1891. doi: 10.3390/v15091891 37766297 PMC10535211

[52] Mangombi JB Roqueplo C Sambou M Dahmani M Mediannikov O Comtet L. Seroprevalence of Crimean-Congo hemorrhagic fever in domesticated animals in northwestern Senegal. Vector Borne Zoonotic Dis. (2020) 20:797–9. doi: 10.1089/vbz.2019.2592, PMID: 32429789

[53] Shepherd AJ Swanepoel R Shepherd SP McGillivray GM Searle LA. Antibody to Crimean-Congo hemorrhagic fever virus in wild mammals from southern Africa. Am J Trop Med Hyg. (1987) 36:133–42. doi: 10.4269/ajtmh.1987.36.133, PMID: 3101526

[54] Atim SA Ashraf S Belij-Rammerstorfer S Ademun AR Vudriko P Nakayiki T. Risk factors for Crimean-Congo Haemorrhagic fever (CCHF) virus exposure in farming communities in Uganda. J Infect. (2022) 85:693–701. doi: 10.1016/j.jinf.2022.09.007, PMID: 36108783 PMC9731351

[55] Swanepoel R Leman PA Burt FJ Jardine J Verwoerd DJ Capua I. Experimental infection of ostriches with Crimean-Congo haemorrhagic fever virus. Epidemiol Infect. (1998) 121:427–32. doi: 10.1017/s0950268898001344, PMID: 9825796 PMC2809542

[56] Shepherd AJ Swanepoel R Leman PA Shepherd SP. Field and laboratory investigation of Crimean-Congo haemorrhagic fever virus (Nairovirus, family Bunyaviridae) infection in birds. Trans R Soc Trop Med Hyg. (1987) 81:1004–7. doi: 10.1016/0035-9203(87)90379-8, PMID: 3140434

[57] Mostafavi E Chinikar S Moradi M Bayat N Meshkat M Fard MK. A case report of crimean Congo hemorrhagic fever in ostriches in Iran. Open Virol J. (2013) 7:81–3. doi: 10.2174/1874357901307010081, PMID: 24015162 PMC3763622

[58] EFSA Panel on Animal Health and Welfare (AHAW). Scientific opinion on geographic distribution of tick-borne infections and their vectors in Europe and the other regions of the Mediterranean Basin. EFSA J. (2010) 8:1723. doi: 10.2903/j.efsa.2010.1723, PMID: 38089469

[59] Bernard C Holzmuller P Bah MT Bastien M Combes B Jori F. Systematic review on Crimean–Congo hemorrhagic fever enzootic cycle and factors favoring virus transmission: special focus on France, an apparently free-disease area in Europe. Front Vet Sci. (2022):9. doi: 10.3389/fvets.2022.932304 PMC9343853 35928117

[60] Darwish MA Hoogstraal H Roberts TJ Ghazi R Amer T. A sero-epidemiological survey for Bunyaviridae and certain other arboviruses in Pakistan. Trans R Soc Trop Med Hyg. (1983) 77:446–50. doi: 10.1016/0035-9203(83)90108-6, PMID: 6415873

[61] Saidi S Casals J Faghih MA. Crimean hemorrhagic fever-Congo (CHF-C) virus antibodies in man, and in domestic and small mammals, in Iran. Am J Trop Med Hyg. (1975) 24:353–7. doi: 10.4269/ajtmh.1975.24.353, PMID: 164135

[62] Burt FJ Swanepoel R Braack LE. Enzyme-linked immunosorbent assays for the detection of antibody to Crimean-Congo haemorrhagic fever virus in the sera of livestock and wild vertebrates. Epidemiol Infect. (1993) 111:547–58. doi: 10.1017/s0950268800057277, PMID: 8270014 PMC2271254

[63] Capek M Literak I Kocianova E Sychra O Najer T Trnka A. Ticks of the *Hyalomma marginatum* complex transported by migratory birds into Central Europe. Ticks Tick Borne Dis. (2014) 5:489–93. doi: 10.1016/j.ttbdis.2014.03.002, PMID: 24877976

[64] Kar S Rodriguez SE Akyildiz G Cajimat MNB Bircan R Mears MC. Crimean-Congo hemorrhagic fever virus in tortoises and *Hyalomma aegyptium* ticks in east Thrace, Turkey: potential of a cryptic transmission cycle. Parasit Vectors. (2020) 13:201. doi: 10.1186/s13071-020-04074-6, PMID: 32307010 PMC7168965

[65] van Eeden PJ Joubert JR van de Wal BW King JB de Kock A Groenewald JH. A nosocomial outbreak of Crimean-Congo haemorrhagic fever at Tygerberg hospital. Part I. Clinical features. S Afr Med J. (1985) 68:711–7. PMID: 4060010

[66] Lindeborg M Barboutis C Ehrenborg C Fransson T Jaenson TGT Lindgren P-E. Migratory birds, ticks, and Crimean-Congo hemorrhagic fever virus. Emerg Infect Dis. (2012) 18:2095–7. doi: 10.3201/eid1812.120718, PMID: 23171591 PMC3557898

[67] Chumakov MP. 30 years of investigation of Crimean hemorrhagic fever (Russian). In: Medical Virology. Tr. Inst Polio Virus Entsef Akad Med Nauk SSSR. (1974) 22:5–18.

[68] Muzammil K Rayyani S Abbas Sahib A Gholizadeh O Naji Sameer H Jwad Kazem T. Recent advances in Crimean-Congo hemorrhagic fever virus detection, treatment, and vaccination: overview of current status and challenges. Biol Proced Online. (2024) 26:20. doi: 10.1186/s12575-024-00244-3, PMID: 38926669 PMC11201903

[69] Febrer-Sendra B Fernández-Soto P García-Bernalt Diego J Crego-Vicente B Negredo A Muñor-Bellido JL. A novel RT-LAMP for the detection of different genotypes of Crimean-Congo Haemorrhagic fever virus in patients from Spain. Int J Mol Sci. (2023) 24:6411. doi: 10.3390/ijms24076411, PMID: 37047384 PMC10094476

[70] Široký P Petrželková KJ Kamler M Mihalca AD Modrý D. *Hyalomma aegyptium* as dominant tick in tortoises of the genus Testudo in Balkan countries, with notes on its host preferences. Exp Appl Acarol. (2007) 40:279–90. doi: 10.1007/s10493-006-9036-z, PMID: 17237970

[71] Shepherd AJ Leman PA Swanepoel R. Viremia and antibody response of small African and laboratory animals to Crimean-Congo hemorrhagic fever virus infection. Am J Trop Med Hyg. (1989) 40:541–7. doi: 10.4269/ajtmh.1989.40.541, PMID: 2499205

[72] Blagoveshchenskaya N Donets MA Zarubina LV Kondratenko VF Kuchin VV. Study of susceptibility to Crimean hemorrhagic fever (CHF) virus in European and long-eared hedgehogs (In Russian). (In English: NAMRU-T985). Tezisy Konf Vop Med Virus. (1975) 2:269–70.

[73] Gonzalez J-P Camicas J-L Cornet J-P Wilson ML. Biological and clinical responses of West African sheep to Crimean-Congo haemorrhagic fever virus experimental infection. Res Virol. (1998) 149:445–55. doi: 10.1016/S0923-2516(99)80013-2, PMID: 9923021

[74] Zarubinsky VY Kondratenko VF Blagoveshchenskaya NM Zarubina LV Kuchin VV. Susceptibility of calves and lambs to Crimean hemorrhagic fever virus. Tezisy Dokl. Vses. Konf. Prir. Ochag. Bolez. Chelov. Zhivot. (1976) 130–131.

[75] Rabinovich VD Milyutin VN Artyushenko AA Buryakov BG Chumakov MP. Possibility of extracting hyperimmune gammaglobulin against CHF from donkey blood sera. (In English: NAMRU3-T1177). Tezisy. (1972) 17:350–1.

[76] Milyutin VN. Experimental infection of horses with Crimean hemorrhagic fever virus. Report I. In Chumakov MP, editor. Arboviruses, mater 16, vol. 2. Moscow: Nauch Sess Inst Polio Virus Entsef (1969). 145–6.

[77] Zivcec M Safronetz D Scott D Robertson S Ebihara H Feldmann H. Lethal Crimean-Congo hemorrhagic fever virus infection in interferon α/β receptor knockout mice is associated with high viral loads, Proinflammatory responses, and coagulopathy. J Infect Dis. (2013) 207:1909–21. doi: 10.1093/infdis/jit061, PMID: 23417661 PMC3654741

[78] Ranadheera C Valcourt EJ Warner BM Poliquin G Rosenke K Frost K. Characterization of a novel STAT 2 knock-out hamster model of Crimean-Congo hemorrhagic fever virus pathogenesis. Sci Rep. (2020) 10:12378. doi: 10.1038/s41598-020-69054-3, PMID: 32704046 PMC7378551

[79] Bereczky S Lindegren G Karlberg H Akerstrom S Klingstrom J Mirazimi A. Crimean-Congo hemorrhagic fever virus infection is lethal for adult type I interferon receptor-knockout mice. J Gen Virol. (2010) 91:1473–7. doi: 10.1099/vir.0.019034-0, PMID: 20164263

[80] Bente DA Alimonti JB Shieh W-J Camus G Ströher U Zaki S. Pathogenesis and immune response of Crimean-Congo hemorrhagic fever virus in a STAT-1 knockout mouse model. J Virol. (2010) 84:11089–100. doi: 10.1128/JVI.01383-10, PMID: 20739514 PMC2953203

[81] Lindquist ME Zeng X Altamura LA Daye SP Delp KL Blancett C. Exploring Crimean-Congo hemorrhagic fever virus-induced hepatic injury using antibody-mediated type I interferon blockade in mice. J Virol. (2018) 92:92. doi: 10.1128/JVI.01083-18, PMID: 30111561 PMC6189508

[82] Haddock E Feldmann F Hawman DW Zivcec M Hanley PW Saturday G. A cynomolgus macaque model for Crimean-Congo haemorrhagic fever. Nat Microbiol. (2018) 3:556–62. doi: 10.1038/s41564-018-0141-7, PMID: 29632370 PMC6717652

[83] Butenko AM Chumakov MP Smirnova SE Vasilenko SM Zavodova TI Tkachenko EA. Isolation of Crimean hemorrhagic fever virus from blood of patients and corpse material (from 1968–1969 investigation data) in Rostov, astrakhan oblast, and Bulgaria. (In English: NAMRU3-T522). Mater 3 oblast Nauchn Prakt Konf. (1970) 6–25.

[84] Fagbami AH Tomori O Fabiyi A Isoun TT. Experimantal Congo virus (Ib -AN 7620) infection in primates. Virologie. (1975) 26:33–7. PMID: 814708

[85] Zeller HG Cornet J-P Camicas J-L. Experimental transmission of Crimean-Congo hemorrhagic fever virus by west African wild ground-feeding birds to *Hyalomma marginatum* rufipes ticks. Am J Trop Med Hyg. (1994) 50:676–81. doi: 10.4269/ajtmh.1994.50.676, PMID: 8024058

[86] Kumar B Manjunathachar HV Ghosh S. A review on Hyalomma species infestations on human and animals and progress on management strategies. Heliyon. (2020) 6:e05675. doi: 10.1016/j.heliyon.2020.e05675, PMID: 33319114 PMC7726666

[87] Berezin VV Chumakov MP Reshetnikov IA Zgurskaya GN. Study of the role of birds in the ecology of Crimean hemorrhagic fever virus. Mater. (1971) 6:94–5.

[88] Erasmus MJ McGillivray GM Gill DE Searle LA Shepherd AJ Swanepoel R. Epidemiologic and clinical features of Crimean-Congo hemorrhagic fever in southern Africa. Am J Trop Med Hyg. (1987) 36:120–32. doi: 10.4269/ajtmh.1987.36.120, PMID: 3101525

[89] Zeller HG Cornet JP Camicas JL. Crimean-Congo haemorrhagic fever virus infection in birds: field investigations in Senegal. Res Virol. (1994) 145:105–9. doi: 10.1016/S0923-2516(07)80012-4, PMID: 8059064

[90] Akuffo R Brandful JAM Zayed A Adjei A Watany N Fahmy NT. Crimean-Congo hemorrhagic fever virus in livestock ticks and animal handler seroprevalence at an abattoir in Ghana. BMC Infect Dis. (2016) 16:324. doi: 10.1186/s12879-016-1660-6, PMID: 27392037 PMC4939019

[91] Causey OR Kemp GE Madbouly MH David-West TS. Congo virus from domestic livestock, African hedgehog, and arthropods in Nigeria. Am J Trop Med Hyg. (1970) 19:846–50. doi: 10.4269/ajtmh.1970.19.846, PMID: 5453910

[92] Naidenova EV Zakharov KS Kartashov MY Agafonov DA Senichkina AM Magassouba N. Prevalence of Crimean-Congo hemorrhagic fever virus in rural areas of Guinea. Ticks Tick Borne Dis. (2020) 11:101475. doi: 10.1016/j.ttbdis.2020.101475, PMID: 32723661

[93] Adjogoua EV Coulibaly-Guindo N Diaha-Kouame CA Diane MK Kouassi RMCKA Coulibaly JT. Geographical distribution of ticks Ixodidae in Côte d’Ivoire: potential reservoir of the Crimean-Congo hemorrhagic fever virus. Vector Borne Zoonotic Dis. (2021) 21:628–34. doi: 10.1089/vbz.2020.2745, PMID: 34037467

[94] Badji A Ndiaye M Gaye A Dieng I Ndiaye EH Dolgova AS. Detection of Crimean-Congo Haemorrhagic fever virus from livestock ticks in northern, central and southern Senegal in 2021. Trop Med Infect Dis. (2023) 8:317. doi: 10.3390/tropicalmed8060317, PMID: 37368735 PMC10303571

[95] Tsapko NV Volynkina AS Evchenko AY Lisitskaya YV Shaposhnikova LI. Detection of Crimean-Congo hemorrhagic fever virus in ticks collected from South Russia. Ticks Tick Borne Dis. (2022) 13:101890. doi: 10.1016/j.ttbdis.2021.101890, PMID: 34953335

[96] Sánchez-Seco MP Sierra MJ Estrada-Peña A Valcárcel F Molina R de Arellano ER. Widespread detection of multiple strains of Crimean-Congo hemorrhagic fever virus in ticks, Spain. Emerg Infect Dis. (2021) 28:394–402. doi: 10.3201/eid2802.211308, PMID: 35076008 PMC8798670

[97] Cuadrado-Matías R Moraga-Fernández A Peralbo-Moreno A Negredo AI Sánchez-Seco MP Ruiz-Fons F. Crimean–Congo haemorrhagic fever virus in questing non-*Hyalomma* spp. ticks in Northwest Spain, 2021. Zoonoses Public Health. (2024) 71:578–83. doi: 10.1111/zph.13130 38590023

[98] Orkun Ö Karaer Z Çakmak A Nalbantoğlu S. Crimean-Congo hemorrhagic fever virus in ticks in Turkey: a broad range tick surveillance study. Infect Genet Evol. (2017) 52:59–66. doi: 10.1016/j.meegid.2017.04.017, PMID: 28433738

[99] Albayrak H Ozan E Kurt M. Molecular detection of Crimean-Congo haemorrhagic fever virus (CCHFV) but not West Nile virus (WNV) in hard ticks from provinces in northern Turkey. Zoonoses Public Health. (2010) 57:e156–60. doi: 10.1111/j.1863-2378.2009.01316.x, PMID: 20163578

[100] Yesilbag K Aydin L Dincer E Alpay G Girisgin AO Tuncer P. Tick survey and detection of Crimean-Congo hemorrhagic fever virus in tick species from a non-endemic area, South Marmara region, Turkey. Exp Appl Acarol. (2013) 60:253–61. doi: 10.1007/s10493-012-9642-x, PMID: 23229492

[101] Shafei E Dayer MS Telmadarraiy Z. Molecular epidemiology of Crimean-Congo hemorrhagic fever virus in ticks in northwest of Iran. J Entomol Zool Stud. (2016) 4:150–4.

[102] de Mera IGF Chaligiannis I Hernández-Jarguín A Villar M Mateos-Hernández L Papa A. Combination of RT-PCR and proteomics for the identification of Crimean-Congo hemorrhagic fever virus in ticks. Heliyon. (2017) 3:e00353. doi: 10.1016/j.heliyon.2017.e00353, PMID: 28736753 PMC5508474

[103] Moming A Yue X Shen S Chang C Wang C Luo T. Prevalence and phylogenetic analysis of Crimean-Congo hemorrhagic fever virus in ticks from different ecosystems in Xinjiang, China. Virol Sin. (2018) 33:67–73. doi: 10.1007/s12250-018-0016-3, PMID: 29524182 PMC6178079

[104] Tekin S Bursali A Mutluay N Keskin A Dundar E. Crimean-Congo hemorrhagic fever virus in various ixodid tick species from a highly endemic area. Vet Parasitol. (2012) 186:546–52. doi: 10.1016/j.vetpar.2011.11.010, PMID: 22119389

[105] Telmadarraiy Z Chinikar S Vatandoost H Faghihi F Hosseini-Chegeni A. Vectors of Crimean Congo hemorrhagic fever virus in Iran. J Arthropod Borne Dis. (2015) 9:137–47. 26623426 PMC4662786

[106] Hekimoglu O Ozer N Ergunay K Ozkul A. Species distribution and detection of Crimean Congo hemorrhagic fever virus (CCHFV) in field-collected ticks in Ankara Province, Central Anatolia, Turkey. Exp Appl Acarol. (2012) 56:75–84. doi: 10.1007/s10493-011-9492-y, PMID: 21910017

[107] Biglari P Chinikar S Belqeiszadeh H Telmadarraiy Z Mostafavi E Ghaffari M. Phylogeny of tick-derived Crimean-Congo hemorrhagic fever virus strains in Iran. Ticks Tick Borne Dis. (2016) 7:1216–21. doi: 10.1016/j.ttbdis.2016.07.012, PMID: 27491289

[108] Telmadarraiy Z Ghiasi SM Moradi M Vatandoost H Eshraghian MR Faghihi F. A survey of Crimean-Congo haemorrhagic fever in livestock and ticks in Ardabil Province, Iran during 2004-2005. Scand J Infect Dis. (2010) 42:137–41. doi: 10.3109/00365540903362501, PMID: 19958240

[109] Kautman M Tiar G Papa A Široký P. AP92-like Crimean-Congo hemorrhagic fever virus in *Hyalomma aegyptium* ticks, Algeria. Emerg Infect Dis. (2016) 22:354–6. doi: 10.3201/eid2202.151528, PMID: 26812469 PMC4734512

[110] Široký P Bělohlávek T Papoušek I Jandzik D Mikulíček P Kubelová M. Hidden threat of tortoise ticks: high prevalence of Crimean-Congo haemorrhagic fever virus in ticks *Hyalomma aegyptium* in the Middle East. Parasit Vectors. (2014) 7:101–4. doi: 10.1186/1756-3305-7-101, PMID: 24618184 PMC3972959

[111] Kasi KK Arnim F Schulz A Rehman A Chudhary A Oneeb M. Crimean-Congo haemorrhagic fever virus in ticks collected from livestock in Balochistan, Pakistan. Transbound Emerg Dis. (2020) 67:1543–52. doi: 10.1111/tbed.13488, PMID: 31961043

[112] Champour M Chinikar S Mohammadi G Razmi G Shah-Hosseini N Khakifirouz S. Molecular epidemiology of Crimean-Congo hemorrhagic fever virus detected from ticks of one humped camels (*Camelus dromedarius*) population in northeastern Iran. J Parasit Dis. (2016) 40:110–5. doi: 10.1007/s12639-014-0458-y, PMID: 27065608 PMC4815851

[113] Shahid MF Yaqub T Ali M Ul-Rahman A Bente DA. Prevalence and phylogenetic analysis of Crimean-Congo hemorrhagic fever virus in ticks collected from Punjab province of Pakistan. Acta Trop. (2021) 218:105892. doi: 10.1016/j.actatropica.2021.105892, PMID: 33753031

[114] Sedaghat M Sarani M Chinikar S Telmadarraiy Z Moghaddam A Azam K. Vector prevalence and detection of Crimean-Congo haemorrhagic fever virus in Golestan Province, Iran. J Vector Borne Dis. (2017) 54:353. doi: 10.4103/0972-9062.225841, PMID: 29460866

[115] Tahmasebi F Ghiasi SM Mostafavi E Moradi M Piazak N Mozafari A. Molecular epidemiology of Crimean-Congo hemorrhagic fever virus genome isolated from ticks of Hamadan province of Iran. J Vector Borne Dis. (2010) 47:211–6. PMID: 21178213

[116] Fakoorziba MR Golmohammadi P Moradzadeh R Moemenbellah-Fard MD Azizi K Davari B. Reverse transcription PCR-based detection of Crimean-Congo hemorrhagic fever virus isolated from ticks of domestic ruminants in Kurdistan province of Iran. Vector Borne Zoonotic Dis. (2012) 12:794–9. doi: 10.1089/vbz.2011.0743, PMID: 22651389

[117] Farhadpour F Telmadarraiy Z Chinikar S Akbarzadeh K Moemenbellah-Fard MD Faghihi F. Molecular detection of Crimean–Congo haemorrhagic fever virus in ticks collected from infested livestock populations in a new endemic area, south of Iran. Trop Med Int Health. (2016) 21:340–7. doi: 10.1111/tmi.12667, PMID: 26758985

[118] Kayedi MH Chinikar S Mostafavi E Khakifirouz S Jalali T Hosseini-Chegeni A. Crimean–Congo hemorrhagic fever virus clade IV (Asia 1) in ticks of Western Iran. J Med Entomol. (2015) 52:1144–9. doi: 10.1093/jme/tjv081, PMID: 26336221

[119] Saghafipour A Mousazadeh-Mojarrad A Arzamani N Telmadarraiy Z Rajabzadeh R Arzamani K. Molecular and seroepidemiological survey on Crimean-Congo hemorrhagic fever virus in northeast of Iran. Med J Islam Repub Iran. (2019) 33:41. doi: 10.47176/mjiri.33.41, PMID: 31456965 PMC6708110

[120] Chumakov MP Bashkirtsev VN Golger EI Dzagurova TK Zavodova TI Konovalov YN. Isolation and identification of Crimean haemorrhagic fever and West Nile fever viruses from ticks collected in Moldavia. (In English: NAMRU3-T1113). Tr. Imt. Polio. Virusn. Entsifalitov Akad. Med. Nauk. SSSR. (1974) 22:45–49.

[121] Pak TP Daniyarov OA Kostyukov MA Bulychev VP Kuima AU. Ecology of Crimean hemorrhagic fever in Tadzhikistan. (In English: NAMRU3-T968). Mater Resp. Simp. Kamenyuki Belovezh Pushoha. Minsk. (1974) 93–94.

[122] Onishchenko GG Tumanova II Vyshemirskiĭ OI Kuhn J Seregin SV Tiunnikov GI. Study of virus contamination of Ixodes ticks in the foci of Crimean-Congo hemorrhagic fever in Kazakhstan and Tajikistan. Zh Mikrobiol Epidemiol Immunobiol. (2005) 1:27–31. PMID: 15773396

[123] Aristova VA Neronov VM Veselovskaya OV Lushchekina AA Kurbanov M. Investigation of Crimean hemorrhagic fever natural foci in south-eastern Turkmenia. Sb Tr Ekol Virus. (1973) 1:115–8.

[124] Williams RJ Al-Busaidy S Mehta FR Maupin GO Wagoner KD Al-Awaidy S. Crimean-Congo haemorrhagic fever: a seroepidemiological and tick survey in the Sultanate of Oman. Trop Med Int Health. (2000) 5:99–106. doi: 10.1046/j.1365-3156.2000.00524.x, PMID: 10747269

[125] Mohammadian M Chinikar S Telmadarraiy Z Vatandoost H Oshaghi MA Hanafi-Bojd AA. Molecular assay on Crimean Congo hemorrhagic fever virus in ticks (Ixodidae) collected from Kermanshah Province, Western Iran. J Arthropod Borne Dis. (2016) 10:381–91. 27308296 PMC4906744

[126] Bryant-Genevier J Bumburidi Y Kazazian L Seffren V Head JR Berezovskiy D. Prevalence of Crimean-Congo hemorrhagic fever virus among livestock and ticks in Zhambyl region, Kazakhstan, 2017. Am J Trop Med Hyg. (2022) 106:1478–85. doi: 10.4269/ajtmh.21-1092, PMID: 35378505 PMC9128673

[127] Fakoorziba MR Naddaf-Sani AA Moemenbellah-Fard MD Azizi K Ahmadnia S Chinikar S. First phylogenetic analysis of a Crimean-Congo hemorrhagic fever virus genome in naturally infected *Rhipicephalus appendiculatus* ticks (Acari: Ixodidae). Arch Virol. (2015) 160:1197–209. doi: 10.1007/s00705-015-2379-1, PMID: 25742932

[128] Kong Y Yan C Liu D Jiang L Zhang G He B. Phylogenetic analysis of Crimean-Congo hemorrhagic fever virus in inner Mongolia, China. Ticks Tick Borne Dis. (2022) 13:101856. doi: 10.1016/j.ttbdis.2021.101856, PMID: 34763306

[129] Smirnova SE Mamaev VI Nepesova NM Filipenko PI VIa K. Study of the circulation of Crimean hemorrhagic fever virus in Turkmenistan. Zh Mikrobiol Epidemiol Immunobiol. (1978) 1:92–7. PMID: 146992

[130] Sultankulova KT Shynybekova GO Kozhabergenov NS Mukhami NN Chervyakova OV Burashev YD. The prevalence and genetic variants of the CCHF virus circulating among ticks in the southern regions of Kazakhstan. Pathogens. (2022) 11:841. doi: 10.3390/pathogens11080841, PMID: 36014962 PMC9414327

[131] Voorhees MA Padilla SL Jamsransuren D Koehler JW Delp KL Adiyadorj D. Crimean-Congo hemorrhagic fever virus, Mongolia, 2013–2014. Emerg Infect Dis. (2018) 24:2202–9. doi: 10.3201/eid2412.180175, PMID: 30457521 PMC6256378

[132] Li Y Yan C Liu D He B Tu C. Seroepidemiological investigation of Crimean-Congo hemorrhagic fever virus in sheep and camels of Inner Mongolia of China. Vector Borne Zoonotic Dis. (2020) 20:461–7. doi: 10.1089/vbz.2019.2529, PMID: 32155395

[133] Schulz A Barry Y Stoek F Pickin MJ Ba A Chitimia-Dobler L. Detection of Crimean-Congo hemorrhagic fever virus in blood-fed Hyalomma ticks collected from Mauritanian livestock. Parasit Vectors. (2021) 14:342. doi: 10.1186/s13071-021-04819-x, PMID: 34187526 PMC8244218

[134] Mohamed RAEH Mohamed N Aleanizy FS Alqahtani FY Al Khalaf A Al-Keridis LA. Investigation of hemorrhagic fever viruses inside wild populations of ticks: one of the pioneer studies in Saudi Arabia. Asian Pac J Trop Dis. (2017) 7:299–303. doi: 10.12980/apjtd.7.2017D6-371

[135] Chisholm K Dueger E Fahmy NT Samaha HAT Zayed A Abdel-Dayem M. Crimean-Congo hemorrhagic fever virus in ticks from imported livestock, Egypt. Emerg Infect Dis. (2012) 18:181–2. doi: 10.3201/eid1801.111071, PMID: 22260737 PMC3310117

[136] Bendary HA Rasslan F Wainwright M Alfarraj S Zaki AM Abdulall AK. Crimean-Congo hemorrhagic fever virus in ticks collected from imported camels in Egypt. Saudi J Biol Sci. (2022) 29:2597–603. doi: 10.1016/j.sjbs.2021.12.043, PMID: 35531170 PMC9072913

[137] Camp JV Weidinger P Ramaswamy S Kannan DO Osman BM Kolodziejek J. Association of Dromedary Camels and Camel Ticks with Reassortant Crimean-Congo hemorrhagic fever virus, United Arab Emirates. Emerg Infect Dis. (2021) 27:2471–4. doi: 10.3201/eid2709.210299, PMID: 34424177 PMC8386785

[138] Bouaicha F Eisenbarth A Elati K Schulz A Ben Smida B Bouajila M. Epidemiological investigation of Crimean-Congo haemorrhagic fever virus infection among the one-humped camels (*Camelus dromedarius*) in southern Tunisia. Ticks Tick Borne Dis. (2021) 12:101601. doi: 10.1016/j.ttbdis.2020.101601, PMID: 33176235

[139] Nabeth P Cheikh DO Lo B Faye O Vall IOM Niang M. Crimean-Congo hemorrhagic fever, Mauritania. Emerg Infect Dis. (2004) 10:2143–9. doi: 10.3201/eid1012.040535, PMID: 15663851 PMC3323392

[140] Chitimia-Dobler L Issa MH Ezalden ME Yagoub IA Abdalla MA Bakhiet AO. Crimean-Congo haemorrhagic fever virus in *Hyalomma impeltatum* ticks from North Kordofan, the Sudan. Int J Infect Dis. (2019) 89:81–3. doi: 10.1016/j.ijid.2019.09.012, PMID: 31521854

[141] Burt FJ Paweska JT Ashkettle B Swanepoel R. Genetic relationship in southern African Crimean-Congo haemorrhagic fever virus isolates: evidence for occurrence of reassortment. Epidemiol Infect. (2009) 137:1302–8. doi: 10.1017/S0950268808001878, PMID: 19161643

[142] Saluzzo JF Digoutte JP Camicas JL Chauvancy G. Crimean-Congo HAEMORRHAGIC fever and Rift Valley fever in south-eastern Mauritania. Lancet. (1985) 325:116. doi: 10.1016/S0140-6736(85)92014-8, PMID: 2857020

[143] Gargili A Midilli K Ergonul O Ergin S Alp HG Vatansever Z. Crimean-Congo hemorrhagic fever in European part of Turkey: genetic analysis of the virus strains from ticks and a Seroepidemiological study in humans. Vector Borne Zoonotic Dis. (2011) 11:747–52. doi: 10.1089/vbz.2010.0030, PMID: 21028961

[144] Kajihara M Simuunza M Saasa N Dautu G Mori-Kajihara A Qiu Y. Serologic and molecular evidence for circulation of Crimean-Congo hemorrhagic fever virus in ticks and cattle in Zambia. PLoS Negl Trop Dis. (2021) 15:e0009452. doi: 10.1371/journal.pntd.0009452, PMID: 34061841 PMC8195391

[145] Goletic T Satrovic L Softic A Omeragic J Goletic S Soldo DK. Serologic and molecular evidence for circulation of Crimean-Congo hemorrhagic fever virus in ticks and cattle in Bosnia and Herzegovina. Ticks Tick Borne Dis. (2022) 13:102004. doi: 10.1016/j.ttbdis.2022.102004, PMID: 35834930

[146] Papa A Velo E Kadiaj P Tsioka K Kontana A Kota M. Crimean-Congo hemorrhagic fever virus in ticks collected from livestock in Albania. Infect Genet Evol. (2017) 54:496–500. doi: 10.1016/j.meegid.2017.08.017, PMID: 28827176

[147] Panayotova E Papa A Trifonova I Christova I. Crimean-Congo hemorrhagic fever virus lineages Europe 1 and Europe 2 in Bulgarian ticks. Ticks Tick Borne Dis. (2016) 7:1024–8. doi: 10.1016/j.ttbdis.2016.05.010, PMID: 27378409

[148] Sherifi K Rexhepi A Berxholi K Mehmedi B Gecaj RM Hoxha Z. Crimean-Congo hemorrhagic fever virus and *Borrelia burgdorferi* sensu lato in ticks from Kosovo and Albania. Front Vet Sci. (2018) 5:38. doi: 10.3389/fvets.2018.00038, PMID: 29560357 PMC5845633

[149] Sherifi K Cadar D Muji S Robaj A Ahmeti S Jakupi X. Crimean-Congo hemorrhagic fever virus clades V and VI (Europe 1 and 2) in ticks in Kosovo, 2012. PLoS Negl Trop Dis. (2014) 8:e3168. doi: 10.1371/journal.pntd.0003168, PMID: 25255381 PMC4177860

[150] Negredo A Habela MÁ Ramírez de Arellano E Diez F Lasala F López P. Survey of Crimean-Congo hemorrhagic fever enzootic focus, Spain, 2011-2015. Emerg Infect Dis. (2019) 25:1177–84. doi: 10.3201/eid2506.180877, PMID: 31107219 PMC6537724

[151] Sang R. Crimean-Congo hemorrhagic fever virus in Hyalommid ticks, Northeastern Kenya. Emerg Infect Dis. (2011) 17:1502–5. doi: 10.3201/eid1708.102064, PMID: 21801635 PMC3381575

[152] Mancuso E Toma L Polci A d’Alessio SG Di Luca M Orsini M. Crimean-Congo hemorrhagic fever virus genome in tick from migratory bird, Italy. Emerg Infect Dis. (2019) 25:1418–20. doi: 10.3201/eid2507.181345, PMID: 31211933 PMC6590740

[153] Sene O Sagne SN Ngom D Diagne MM Badji A Khoulé A. Emergence of Crimean-Congo hemorrhagic fever virus in eastern Senegal in 2022. Viruses. (2024) 16:315. doi: 10.3390/v16020315, PMID: 38400090 PMC10891565

[154] Simo Tchetgna H Yousseu FS Cosset F-L de Freitas NB Kamgang B McCall PJ. Molecular and serological evidence of Crimean-Congo hemorrhagic fever orthonairovirus prevalence in livestock and ticks in Cameroon. Front Cell Infect Microbiol. (2023) 13:13. doi: 10.3389/fcimb.2023.1132495, PMID: 37056704 PMC10086150

[155] Jarosław P Łukasz G Agnieszka F Mirosław W Janusz P. Vector and serologic survey for Crimean–Congo hemorrhagic fever virus in Poland. Vector Borne Zoonotic Dis. (2017) 17:510–3. 10.1089/vbz.2016.2075 28514225

[156] Atim SA Niebel M Ashraf S Vudriko P Odongo S Balinandi S. Prevalence of Crimean-Congo haemorrhagic fever in livestock following a confirmed human case in Lyantonde district, Uganda. Parasit Vectors. (2023) 16:7. doi: 10.1186/s13071-022-05588-x, PMID: 36611216 PMC9824997

[157] Wampande EM Waiswa P Allen DJ Hewson R Frost SDW Stubbs SCB. Phylogenetic characterization of Crimean-Congo hemorrhagic fever virus detected in African blue ticks feeding on cattle in a Ugandan abattoir. Microorganisms. (2021) 9:9. doi: 10.3390/microorganisms9020438, PMID: 33672497 PMC7923759

[158] Chiuya T Masiga DK Falzon LC Bastos ADS Fèvre EM Villinger J. Tick-borne pathogens, including Crimean-Congo haemorrhagic fever virus, at livestock markets and slaughterhouses in western Kenya. Transbound Emerg Dis. (2021) 68:2429–45. doi: 10.1111/tbed.13911, PMID: 33142046 PMC8359211

[159] Mhamadi M Badji A Dieng I Gaye A Ndiaye EH Ndiaye M. Crimean-Congo hemorrhagic fever virus survey in humans, ticks, and livestock in Agnam (northeastern Senegal) from February 2021 to march 2022. Trop Med Infect Dis. (2022) 7:324. doi: 10.3390/tropicalmed7100324, PMID: 36288065 PMC9610667

[160] Telmadarraiy Z Moradi AR Vatandoost H Mostafavi E Oshaghi MA Zahirnia AH. Crimean-Congo hemorrhagic fever: a seroepidemiological and molecular survey in Bahar, Hamadan Province of Iran. Asian J Anim Vet Adv. (2008) 3:321–327. doi: 10.3923/ajava.2008.321.327

[161] Jafari A Rasekh M Saadati D Faghihi F Fazlalipour M. Molecular detection of Crimean-Congo Haemorrhagic fever (CCHF) virus in hard ticks from South Khorasan, east of Iran. J Vector Borne Dis. (2022) 59:241–5. doi: 10.4103/0972-9062.342400, PMID: 36511040

[162] Darwish MA Imam IZ Omar FM Hoogstraal H. Results of a preliminary seroepidemiological survey for Crimean-Congo hemorrhagic fever virus in Egypt. Acta Virol. (1978) 22:77. 25013

[163] Kiwan P Masse S Piorkowski G Ayhan N Gasparine M Vial L. Crimean-Congo hemorrhagic fever virus in ticks collected from cattle, Corsica, France, 2023. Emerg Infect Dis. (2024) 30:1036–9. doi: 10.3201/eid3005.231742, PMID: 38666687 PMC11060454

[164] Mohamed M Said A-R Murad A Graham R. A serological survey of Crimean-Congo haemorrhagic fever in animals in the Sharkia governorate of Egypt. Vet Ital. (2008) 44:513–7. PMID: 20405447

[165] Lugaj A Koni M Mertens M Groschup M Berxholi K. Serological survey of Crimean-Congo hemorrhagic fever virus in cattle in Berat and Kolonje, Albania. Albanian J Agric Sci. (2014) 13:325–8.

[166] Lugaj A Mertens M Groschup MH Bërxholi K. Serological survey of CCHFV in cattle in 10 regions of Albania. Int J Res Appl Nat Soc Sci. (2014) 2:55–60.

[167] Chumakov MP Ismailova ST Rubin SG Smirnova SE Zgurskaya GN Khankishiev AS. Detection of Crimean hemorrhagic fever foci in Azerbaijan SSR from results from serological investigations of domestic animals. Trudy Inst Polio Virus Entsef Akad Med Nauk SSSR. (1970) 18:120–2.

[168] Matevosyan KS Semashko I Rubin S Chumakov M. Antibodies to CHF virus in human and cattle blood sera from Armenian SSR (NAMRU-T939). Tr Inst Polio Virus Entsef Akad Med Nauk SSSR. (1974) 22:173–5.

[169] Horváth LB. Incidence of antibodies to Crimean haemorrhagic fever in animals (author’s transl). Acta Microbiol Acad Sci Hung. (1975) 22:61–3. PMID: 803341

[170] Horton KC Wasfy M Samaha H Abdel-Rahman B Safwat S Abdel Fadeel M. Serosurvey for zoonotic viral and bacterial pathogens among slaughtered livestock in Egypt. Vector Borne Zoonotic Dis. (2014) 14:633–9. doi: 10.1089/vbz.2013.1525, PMID: 25198525 PMC4676263

[171] Gergova I Kamarinchev B. Comparison of the prevalence of Crimean-Congo hemorrhagic fever virus in endemic and non-endemic Bulgarian locations. J Vector Borne Dis. (2013) 50:265–70. doi: 10.4103/0972-9062.126410, PMID: 24499848

[172] Yadav PD Gurav YK Mistry M Shete AM Sarkale P Deoshatwar AR. Emergence of Crimean-Congo hemorrhagic fever in Amreli District of Gujarat state, India, June to July 2013. Int J Infect Dis. (2014) 18:97–100. doi: 10.1016/j.ijid.2013.09.019, PMID: 24211848

[173] Chumakov M Smirnova S. Detection of antibodies to CHF in wild and domestic animal blood sera from Iran and Africa. (In English: NAMRU T1072). Tezisy 17 Nauch Sees Inst Posvyashch Aktual Probl Virus Profil Virus Zabolev. (1972) 367–8.

[174] Mostafavi E Haghdoost A Khakifirouz S Chinikar S. Spatial analysis of Crimean Congo hemorrhagic fever in Iran. Am J Trop Med Hyg. (2013) 89:1135–41. doi: 10.4269/ajtmh.12-0509, PMID: 24166038 PMC3854891

[175] Chinikar S Ghiasi SM Naddaf S Piazak N Moradi M Razavi MR. Serological evaluation of Crimean-Congo hemorrhagic fever in humans with high-risk professions living in enzootic regions of Isfahan province of Iran and genetic analysis of circulating strains. Vector Borne Zoonotic Dis. (2012) 12:733–8. doi: 10.1089/vbz.2011.0634, PMID: 22217167 PMC3438802

[176] Fajs L Humolli I Saksida A Knap N Jelovšek M Korva M. Prevalence of Crimean-Congo hemorrhagic fever virus in healthy population, livestock and ticks in Kosovo. PLoS One. (2014) 9:e110982. doi: 10.1371/journal.pone.0110982, PMID: 25393542 PMC4230912

[177] Semashko I Dobritsa P Bashkirtsev V Chumakov M. Results from investigating blood sera from healthy persons, animals, and birds collected in southern Kazakhstan for antibodies to CHF-Congo virus. (In English: NAMRU-T1128). Mater 9 Simp Ekol Virus. (1975) 43–44.

[178] Hassanein KM el-Azazy OM Yousef HM. Detection of Crimean-Congo haemorrhagic fever virus antibodies in humans and imported livestock in Saudi Arabia. Trans R Soc Trop Med Hyg. (1997) 91:536–7. doi: 10.1016/s0035-9203(97)90014-6, PMID: 9463660

[179] Umoh JU Ezeokoli CD Ogwu D. Prevalence of antibodies to Crimean-haemorrhagic fever-Congo virus in cattle in northern Nigeria. Int J Zoonoses. (1983) 10:151–4. PMID: 6427128

[180] Mariner JC Morrill J Ksiazek TG. Antibodies to hemorrhagic fever viruses in domestic livestock in Niger: Rift Valley fever and Crimean-Congo hemorrhagic fever. Am J Trop Med Hyg. (1995) 53:217–21. doi: 10.4269/ajtmh.1995.53.217, PMID: 7573699

[181] Chunikhin SP Chumakov MP Butenko AM Smirnova SE Taufflieb R Camicas J-L. Results from investigating human and domestic and wild animal blood sera in the Senegal Republic (Western Africa) for antibodies to crimean hemorrhagic fever virus. Nauchn Sess Inst Polio Virus Entsefalitov (Moscow). (1969) 2:158–60.

[182] Berezin V Chumakov M Rubin S Stolbov D Butenko A Bashkirtsev V. Contribution to the ecology of Crimean hemorrhagic fever virus in the lower Volga River. (In English: NAMRU-T836). Arboviruses. (1969) 2:120–2.

[183] Karinskaya GA Chumakov MP Butenko AM Badalov ME Rubin SG. Investigation of antibodies to Crimean hemorrhagic fever virus in animal blood samples from Rostov oblast. A translation of “Crimean hemorrhagic fever”: papers from the third regional workshop at Rostov-on-Don in May 1970. BioOne. (1974) 9:147. doi: 10.4182/KGVP3013.9-3.147

[184] Kuchin VV Yanovich TD Butenko AM Kirsanova KS. Serological examination for antibodies to CHF virus in domestic animals of Rostov oblast. A translation of “Crimean hemorrhagic fever”: papers from the third regional workshop at Rostov-on-Don in may 1970. BioOne. (1974) 9:149. doi: 10.4182/KGVP3013.9-3.149

[185] Mertens M Vatansever Z Mrenoshki S Krstevski K Stefanovska J Djadjovski I. Circulation of Crimean-Congo hemorrhagic fever virus in the former Yugoslav Republic of Macedonia revealed by screening of cattle sera using a novel enzyme-linked immunosorbent assay. PLoS Negl Trop Dis. (2015) 9:e0003519. doi: 10.1371/journal.pntd.0003519, PMID: 25742017 PMC4351108

[186] Khan AS Maupin GO Rollin PE Noor AM Shurie HH Shalabi AG. An outbreak of Crimean-Congo hemorrhagic fever in the United Arab Emirates, 1994-1995. Am J Trop Med Hyg. (1997) 57:519–25. doi: 10.4269/ajtmh.1997.57.519, PMID: 9392589

[187] Swanepoel R Shepherd AJ Leman PA Shepherd SP. Investigations following initial recognition of Crimean-Congo haemorrhagic fever in South Africa and the diagnosis of 2 further cases. S Afr Med J. (1985) 68:638–41. PMID: 3933132

[188] Kirya B Semenov B Tretiyakov A Gromashevsky V Madzhomba E. Preliminary report on investigating animal sera from East Africa for antibodies to Congo virus by the agar gel diffusion and precipitation method. NAMRU-T1073 Tezisy. (1972) 17:368–9.

[189] Tuncer P Yesilbag K Alpay G Dincer E Girisgin AO Aydin L. Crimean-Congo Hemorrhagic Fever infection in domestic animals in Marmara region, Western Turkey. Ankara Univ Vet Fak Derg. (2014) 61:49–53.

[190] Blanco-Penedo I Obanda V Kingori E Agwanda B Ahlm C Lwande OW. Seroepidemiology of Crimean-Congo hemorrhagic fever virus (CCHFV) in cattle across three livestock pastoral regions in Kenya. Dairy. (2021) 2:425–34. doi: 10.3390/dairy2030034

[191] Deézsi-Magyar N Dénes B Novák B Zsidei G Déri D Henczkó J. First broad-range serological survey of Crimean-Congo hemorrhagic fever among Hungarian livestock. Viruses. (2024) 16:875. doi: 10.3390/v16060875, PMID: 38932166 PMC11209279

[192] El Ghassem A Apolloni A Vial L Bouvier R Bernard C Khayar MS. Risk factors associated with Crimean-Congo hemorrhagic fever virus circulation among human, livestock and ticks in Mauritania through a one health retrospective study. BMC Infect Dis. (2023) 23:764. doi: 10.1186/s12879-023-08779-8, PMID: 37932678 PMC10626674

[193] Mohammad HHB Abdulmajeed HA Hammad MH Mohamed SA Saif SA Salim AL. Cross-sectional survey of Crimean-Congo hemorrhagic fever virus in the sultanate of Oman. J Vet Med Anim Health. (2016) 8:44–9. doi: 10.5897/JVMAH2016.0472

[194] Zouaghi K Bouattour A Aounallah H Surtees R Krause E Michel J. First serological evidence of Crimean-Congo hemorrhagic fever virus and Rift Valley fever virus in ruminants in Tunisia. Pathogens. (2021) 10:769. doi: 10.3390/pathogens10060769, PMID: 34207423 PMC8234966

[195] Sas MA Mertens M Isselmou E Reimer N El Mamy BO Doumbia B. Crimean-Congo hemorrhagic fever virus-specific antibody detection in cattle in Mauritania. Vector Borne Zoonotic Dis. (2017) 17:582–7. doi: 10.1089/vbz.2016.2084, PMID: 28605299

[196] Schulz A Barry Y Stoek F Ba A Schulz J Haki ML. Crimean-Congo hemorrhagic fever virus antibody prevalence in Mauritanian livestock (cattle, goats, sheep and camels) is stratified by the animal’s age. PLoS Negl Trop Dis. (2021) 15:e0009228. doi: 10.1371/journal.pntd.0009228, PMID: 33844691 PMC8081336

[197] Ngom D Khoulé A Faye ET Sène O Diop SM Sagne SN. Crimean-Congo haemorrhagic fever outbreak in northern Senegal in 2022: prevalence of the virus in livestock and ticks, associated risk factors and epidemiological implications. Zoonoses Public Health. (2024) 71:696–707. doi: 10.1111/zph.13136, PMID: 38627964 PMC11368619

[198] Mourya DT Yadav PD Shete AM Sathe PS Sarkale PC Pattnaik B. Cross-sectional Serosurvey of Crimean-Congo hemorrhagic fever virus IgG in livestock, India, 2013–2014. Emerg Infect Dis. (2015) 21:1837–9. doi: 10.3201/eid2110.141961, PMID: 26402332 PMC4593432

[199] Özüpak T Albayrak H. Molecular detection of crimean-Congo hemorrhagic fever virus (CCHFV)in tick samples but not in blood and milk samples of domestic ruminant species (cattle, sheep and goat) in northern Turkey. Pol J Vet Sci. (2020) 23:651–3. doi: 10.24425/pjvs.2020.135809, PMID: 33480505

[200] Tekelioğlu BK Ozan E Ütük AE Atli AH Albayrak H Elsabagh M. Seroepidemiological survey of the Crimean-Congo hemorrhagic fever virus (CCHFV) infection amongst domestic ruminants in Adana province, East Mediterranean, Turkey. J Adv VetBio Sci Tech. (2021) 6:228–38. doi: 10.31797/vetbio.997150

[201] Mourya DT Yadav PD Shete A Majumdar TD Kanani A Kapadia D. Serosurvey of Crimean-Congo hemorrhagic fever virus in domestic animals, Gujarat, India, 2013. Vector Borne Zoonotic Dis. (2014) 14:690–2. doi: 10.1089/vbz.2014.1586, PMID: 25229708

[202] Shanmugam J Smirova S Chumakov M. Detection of antibodies to CHF-Congo viruses in human and domestic animal blood sera in India. Tr Inst Polio Virus Entsef. (1973). 21: 149–152.

[203] Papa A Velo E Papadimitriou E Cahani G Kota M Bino S. Ecology of the Crimean-Congo hemorrhagic fever endemic area in Albania. Vector Borne Zoonotic Dis. (2009) 9:713–6. doi: 10.1089/vbz.2008.0141, PMID: 19402760

[204] Kasi KK Sas MA Sauter-Louis C von Arnim F Gethmann JM Schulz A. Epidemiological investigations of Crimean-Congo haemorrhagic fever virus infection in sheep and goats in Balochistan, Pakistan. Ticks Tick Borne Dis. (2020) 11:101324. doi: 10.1016/j.ttbdis.2019.101324, PMID: 31757688

[205] Papa A Chaligiannis I Kontana N Sourba T Tsioka K Tsatsaris A. A novel AP92-like Crimean-Congo hemorrhagic fever virus strain, Greece. Ticks Tick Borne Dis. (2014) 5:590–3. doi: 10.1016/j.ttbdis.2014.04.008, PMID: 24953797

[206] Taraku A Bizhga B Korro K Berxholi K Lugaj A Groschup MH. Sheep as the hosts of the CCHF and tick in Kosovo. Anglisticum. (2015) 4:151–6.

[207] Mahzounieh M Dincer E Faraji A Akin H Akkutay AZ Ozkul A. Relationship between Crimean-Congo hemorrhagic fever virus strains circulating in Iran and Turkey: possibilities for Transborder transmission. Vector Borne Zoonotic Dis. (2012) 12:782–5. doi: 10.1089/vbz.2011.0928, PMID: 22925023 PMC3438804

[208] Khamassi Khbou M Romdhane R Bouaicha Zaafouri F Bouajila M Sassi L Appelberg SK. Presence of antibodies to Crimean Congo haemorrhagic fever virus in sheep in Tunisia, North Africa. Vet Med Sci. (2021) 7:2323–9. doi: 10.1002/vms3.597, PMID: 34390548 PMC8604105

[209] Ceianu CS Panculescu-Gatej RI Coudrier D Bouloy M. First serologic evidence for the circulation of Crimean-Congo hemorrhagic fever virus in Romania. Vector Borne Zoonotic Dis. (2012) 12:718–21. doi: 10.1089/vbz.2011.0768, PMID: 22897346

[210] Schuster I Chaintoutis SC Dovas CI Groschup MH Mertens M. Detection of Crimean-Congo hemorrhagic fever virus-specific IgG antibodies in ruminants residing in central and Western Macedonia, Greece. Ticks Tick Borne Dis. (2017) 8:494–8. doi: 10.1016/j.ttbdis.2017.02.009, PMID: 28286143

[211] Morrill JC Soliman AK Imam IZ Botros BA Moussa MI Watts DM. Serological evidence of Crimean-Congo haemorrhagic fever viral infection among camels imported into Egypt. J Trop Med Hyg. (1990) 93:201–4. PMID: 2112203

[212] Champour M Mohammadi G Chinikar S Razmi G Shah-Hosseini N Khakifirouz S. Seroepidemiology of Crimean-Congo hemorrhagic fever virus in one-humped camels (*Camelus dromedarius*) population in northeast of Iran. J Vector Borne Dis. (2014) 51:62–5. doi: 10.4103/0972-9062.130163, PMID: 24717205

[213] Champour M Chinikar S Mohammadi G Razmi G Mostafavi E Shah-Hosseini N. Crimean-Congo hemorrhagic fever in the one-humped camel (*Camelus dromedarius*) in east and northeast of Iran. J Arthropod Borne Dis. (2016) 10:168–77. PMID: 27308275 PMC4906756

[214] Suliman HM Adam IA Saeed SI Abdelaziz SA Haroun EM Aradaib IE. Crimean Congo hemorrhagic fever among the one-humped camel (Camelus dromedaries) in Central Sudan. Virol J. (2017) 14:147. doi: 10.1186/s12985-017-0816-3, PMID: 28774342 PMC5543554

[215] Camp JV Kannan DO Osman BM Shah MS Howarth B Khafaga T. Crimean-Congo hemorrhagic fever virus Endemicity in United Arab Emirates, 2019. Emerg Infect Dis. (2020) 26:1019–21. doi: 10.3201/eid2605.191414, PMID: 32097111 PMC7181925

[216] Khalafalla AI Li Y Uehara A Hussein NA Zhang J Tao Y. Identification of a novel lineage of Crimean-Congo haemorrhagic fever virus in dromedary camels, United Arab Emirates. J Gen Virol. (2021) 102:102. doi: 10.1099/jgv.0.001473, PMID: 33231536 PMC8749806

[217] Zarubinsky VY Klisenko GA Kuchin VV Timchenko VV Shanoyan NK. Application of the indirect hemagglutination inhibition test for serological investigation of Crimean hemorrhagic fever focus in Rostov oblast. In: English, (NAMRU3-T1145) Sb Tr Inst virus Im DI Ivanov Akad med Nauk SSSR, (1975) 2:73–7.

[218] Smirnova S Zgurskaya G Nepesova N Pak P Chumakov M Chunikhin S. Examination of animal blood samples in Central Asia for antibodies to Crimean hemorrhagic fever virus (CHF) (in Russian) (in English, NAMRU3-T820). In: Mater 16 Nauch Sess Inst Polio Virus Entsef. Moscow, October. (1969) 2:146–7.

[219] Arata AA. The importance of small mammals in public health. Int Biol Program. (1975) 5:349–59.

[220] Németh V Oldal M Egyed L Gyuranecz M Erdélyi K Kvell K. Serologic evidence of Crimean-Congo hemorrhagic fever virus infection in Hungary. Vector Borne Zoonotic Dis. (2013) 13:270–2. doi: 10.1089/vbz.2012.1011, PMID: 23421895

[221] Chunikhin SP Chumakov MP Smirnova SE Pak TP Pavlovich AN Kuima AU. Division into biocenotic groups of mammals and ixodid ticks in Crimean hemorrhagic foci of southern Central Asia. In: English, (NAMRU3-T821) Mater 16 Nauch Sess Inst Polio Virus Entsef. Moscow, October. (1969) 156–7.

[222] Földes F Madai M Németh V Zana B Papp H Kemenesi G. Serologic survey of the Crimean-Congo haemorrhagic fever virus infection among wild rodents in Hungary. Ticks Tick Borne Dis. (2019) 10:101258. doi: 10.1016/j.ttbdis.2019.07.002, PMID: 31302067

[223] Sonenshine DE Mather TN. Ecological dynamics of tick-borne zoonoses. Oxford, England, United Kingdom: Oxford University Press (1994).

[224] Msimang V Weyer J le Roux C Kemp A Burt FJ Tempia S. Risk factors associated with exposure to Crimean-Congo haemorrhagic fever virus in animal workers and cattle, and molecular detection in ticks, South Africa. PLoS Negl Trop Dis. (2021) 15:e0009384. doi: 10.1371/journal.pntd.0009384, PMID: 34048430 PMC8162673

[225] Mostafavi E Pourhossein B Esmaeili S Bagheri Amiri F Khakifirouz S Shah-Hosseini N. Seroepidemiology and risk factors of Crimean-Congo hemorrhagic fever among butchers and slaughterhouse workers in southeastern Iran. Int J Infect Dis. (2017) 64:85–9. doi: 10.1016/j.ijid.2017.09.008, PMID: 28935247

[226] Hawman DW Feldmann H. Crimean-Congo haemorrhagic fever virus. Nat Rev Microbiol. (2023) 21:463–77. doi: 10.1038/s41579-023-00871-9, PMID: 36918725 PMC10013989

